# Rotavirus Activates Lymphocytes from Non-Obese Diabetic Mice by Triggering Toll-Like Receptor 7 Signaling and Interferon Production in Plasmacytoid Dendritic Cells

**DOI:** 10.1371/journal.ppat.1003998

**Published:** 2014-03-27

**Authors:** Jessica A. Pane, Nicole L. Webster, Barbara S. Coulson

**Affiliations:** Department of Microbiology and Immunology, The University of Melbourne, Parkville, Victoria, Australia; Cincinnati Children's Hospital Medical Center, United States of America

## Abstract

It has been proposed that rotavirus infection promotes the progression of genetically-predisposed children to type 1 diabetes, a chronic autoimmune disease marked by infiltration of activated lymphocytes into pancreatic islets. Non-obese diabetic (NOD) mice provide a model for the human disease. Infection of adult NOD mice with rhesus monkey rotavirus (RRV) accelerates diabetes onset, without evidence of pancreatic infection. Rather, RRV spreads to the pancreatic and mesenteric lymph nodes where its association with antigen-presenting cells, including dendritic cells, induces cellular maturation. RRV infection increases levels of the class I major histocompatibility complex on B cells and proinflammatory cytokine expression by T cells at these sites. In autoimmunity-resistant mice and human mononuclear cells from blood, rotavirus-exposed plasmacytoid dendritic cells contribute to bystander polyclonal B cell activation through type I interferon expression. Here we tested the hypothesis that rotavirus induces bystander activation of lymphocytes from NOD mice by provoking dendritic cell activation and proinflammatory cytokine secretion. NOD mouse splenocytes were stimulated with rotavirus and assessed for activation by flow cytometry. This stimulation activated antigen-presenting cells and B cells independently of virus strain and replicative ability. Instead, activation depended on virus dose and was prevented by blockade of virus decapsidation, inhibition of endosomal acidification and interference with signaling through Toll-like receptor 7 and the type I interferon receptor. Plasmacytoid dendritic cells were more efficiently activated than conventional dendritic cells by RRV, and contributed to the activation of B and T cells, including islet-autoreactive CD8^+^ T cells. Thus, a double-stranded RNA virus can induce Toll-like receptor 7 signaling, resulting in lymphocyte activation. Our findings suggest that bystander activation mediated by type I interferon contributes to the lymphocyte activation observed following RRV infection of NOD mice, and may play a role in diabetes acceleration by rotavirus.

## Author Summary

Understanding how viruses contribute to type 1 diabetes development is vital for disease prevention. Infection of children at-risk of diabetes with the gastrointestinal pathogen rotavirus is associated with increased immune responses to pancreatic islets, leading to the proposal that rotavirus infection may accelerate progression to diabetes. In a mouse model, we showed previously that rotavirus accelerates diabetes onset, in conjunction with virus spread to the lymph nodes, draining the intestine and pancreas. At these sites, rotavirus associates with antigen-presenting cells of the immune system, including dendritic cells, leading to their maturation, and induces the activation of B and T cells. Here we use this mouse model to define the contribution of rotavirus-exposed antigen-presenting cells to the activation of neighboring B and T cells. We found that rotavirus-exposed dendritic cells induce B and T cell activation through secretion of type I interferon. Activation of these dendritic cells depends on recognition of viral RNA by Toll-like receptor 7. Our studies suggest that this mechanism of B and T cell activation may occur in RRV-infected mice and contribute to their accelerated diabetes development. A similar mechanism may be involved in the enhanced islet autoantibody responses of children following rotavirus infection.

## Introduction

Type 1 diabetes is a chronic autoimmune disease marked by infiltration of immune cells into pancreatic islets and destruction of insulin-secreting β cells [1]. Diabetes development is associated with specific high-risk human leukocyte antigen haplotypes [2]. However, genetic susceptibility cannot explain the discordance between monozygotic twins, seasonality of disease, rising incidence and trend towards a younger age of onset [3]. Environmental factors such as dietary proteins, intestinal microbiota and virus infections are implicated in diabetes development [4], [5]. Widely studied virus modulators of diabetes include enteroviruses [6], particularly coxsackieviruses [7]. In addition, rotavirus infection in children genetically at-risk of type 1 diabetes is associated with increased islet autoantibody levels and has been proposed to accelerate progression to diabetes [8], [9].

Virus-mediated acceleration of diabetes development is proposed to occur by three distinct but not mutually-exclusive mechanisms: direct pancreatic infection, T cell molecular mimicry and bystander activation [10]. In the absence of direct β cell infection and lysis, pancreatic infection and molecular mimicry would lead to T cell activation via antigen presentation on the major histocompatibility complex (MHC). However, bystander activation would involve polyclonal lymphocyte activation by cytokine-secreting antigen-presenting cells (APCs). Thus, bystander activation is not antigen-specific, and depends on the presence of autoreactive B and T cells. The host sites targeted by virus and the timing of infection in relation to the degree of islet autoimmunity would influence the likelihood of bystander activation.

Rhesus monkey rotavirus (RRV) infection in adult non-obese diabetic (NOD) mice induces early diabetes onset, but this does not involve pancreatic infection [11]. Instead, this diabetes acceleration is associated with a Th1-biased antibody and cytokine response [12]. As rotavirus infection accelerates diabetes only in mice with established insulitis, the presence of autoreactive cells is required for this process [11], [13]. Infectious RRV is present in the mesenteric lymph nodes (MLN) of NOD mice, where its APC association correlates with increased MHC expression [12]. The pancreatic lymph nodes (PLN), where islet-autoreactive cells accumulate, also contain infectious RRV [12]. Dendritic cells (DC) and T cells in the PLN of RRV-infected NOD mice express increased pro-inflammatory cytokine levels, and B cells show increased MHC I expression [14]. Rotavirus does not associate with B or T cells in MLN or PLN of NOD mice [12]. We have proposed that RRV-activated APC induce bystander activation of lymphocytes, which contributes to diabetes acceleration following RRV infection of NOD mice [14]. Infection with murine rotavirus, but not porcine rotavirus CRW-8, also accelerates diabetes development in NOD mice [11], [12], [14].

The potential for bystander activation following exposure to rotavirus has been demonstrated previously in non-autoimmune systems. Polyclonal B cell activation occurs after exposure of isolated murine splenocytes and human peripheral blood mononuclear cells (PBMC) to RRV, and is independent of virus replication [15], [16]. B cell activation is independent of the rotavirus or mouse strain, and prevented by rotavirus outer capsid removal or blockade with neutralising antibodies to outer capsid protein VP7 [15]. Recently, RRV stimulation of human plasmacytoid DC (pDC), and their secretion of type I interferon (IFN), was shown to be required for human B cell activation [17]. Type I IFN signaling and the presence of pDC also contributed to B cell activation in C57BL/6 mice infected with murine rotavirus [17]. RRV-exposed but not productively infected human pDC secrete the type I IFN, IFNα [18]. This pDC activation requires rotavirus structural proteins and its double-stranded (ds) RNA genome. Depletion of human pDC in PBMC cultures reduces the frequency of rotavirus-specific T cells expressing IFNγ after rotavirus exposure, so pDC also are important for T cell activation [19]. As IFNα stimulation is not T cell receptor-specific, it is likely that pDC depletion also reduces the activation of T cells specific for antigens other than rotavirus. Therefore, IFNα production by RRV-exposed pDC contributes to non-specific B and T cell activation in non-autoimmune human and mouse model systems.

In naive NOD mice, the numbers of IFNα-producing pDC increase in PLN at 3 to 4 weeks of age, and antibody blockade of the type I IFN receptor (IFNAR) prior to this age delays and significantly reduces the incidence of diabetes [20], [21]. Blockade of IFNα expression by pDC also reduces the frequency and activation of islet-specific CD8^+^ T cells in PLN [22]. Furthermore, pDC depletion significantly reduces diabetes incidence in NOD mice [22]. As IFNα expression by pDC appears to play an important role in diabetes development, it is reasonable to propose that augmentation of pDC responses following rotavirus infection might accelerate diabetes development. Supporting this, the detection of IFNα and coxsackievirus mRNA in the blood of diabetic children is correlated [23]. In mice, both RRV-induced diabetes acceleration and type I IFN responses are associated with the production of a Th1-biased antibody response [12], [24].

The aim of this study was to determine if exposure of immune cells from NOD mice to rotavirus induces polyclonal bystander activation of lymphocytes, and whether this occurs through virus activation of pDC and type I IFN production. We found that rotavirus-stimulated splenocytes exhibited dose-dependent APC and B cell activation that was independent of virus replication or strain. This activation was associated with IFNα secretion and prevented by rotavirus treatment with VP7 neutralising antibody, inhibition of endosomal acidification or TLR7 signaling and blockade of signaling through the IFNAR. Importantly, pDC (and to a lesser extent, conventional DC (cDC)) were shown to contribute to B and T lymphocyte activation following RRV exposure. It was further demonstrated that rotavirus induces the activation of islet autoreactive T cells. These data provide evidence that bystander activation may be an important mechanism for lymphocyte activation during RRV-mediated diabetes acceleration in NOD mice.

## Results

### APCs and B cells were activated following exposure of NOD splenocytes to rotavirus

**Figure 1:**
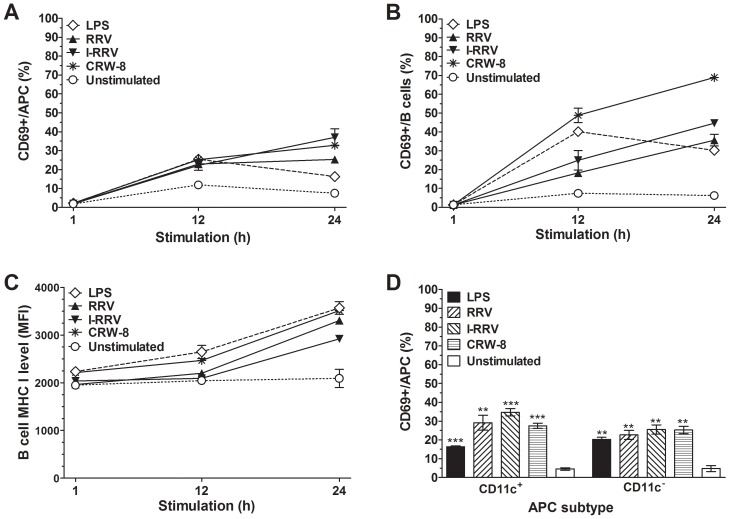
Rotavirus simulation of splenocytes induced APC and B cell activation. Cells (5×10^5^) isolated from 12 week-old naive female NOD mice were cultured in the presence of 100 ng/ml LPS, RRV, I-RRV, CRW-8 or left unstimulated. The proportions of activated (CD69^+^) live cells at 1 h, 12 h and 24 h after treatment were determined for (A) APC (CD3^−^CD19^−^MHCII^+^) and (B) B cells (CD3^−^CD19^+^). (C) Surface MHC I level (mean fluorescence intensity) on B cells at 1 h, 12 h and 24 h of stimulation. (D) Proportions of CD11c^+^ APCs and CD11c^−^ APCs expressing CD69 at 24 h after stimulation. Data are derived from one experiment and are representative of two independent experiments. Error bars indicate the mean ± SEM of 3 replicates. ** p<0.01 and *** p<0.001 compared with the respective unstimulated control.

The activation status of NOD mouse splenocytes cultured in the presence of rotavirus was analysed. Splenocytes were stimulated with RRV, I-RRV and CRW-8, of which only RRV accelerates diabetes onset in diabetes-prone mice [11], [12]. As expected, the proportion of activated (CD69^+^) APCs and B cells increased following control stimulation with bacterial lipopolysaccharide (LPS) for 12 h or 24 h (Figure 1A, 1B). The proportion of APCs (Figure 1A) and B cells (Figure 1B) expressing CD69 was significantly increased over unstimulated controls after stimulation with RRV, I-RRV or CRW-8 for 12 h (p≤0.021 and p≤0.029, respectively) and 24 h (p≤0.023 and p≤0.011, respectively). No APC or B cell activation was detected at 1 h after rotavirus exposure (p>0.05). The proportion of activated APCs and B cells increased with the duration of rotavirus exposure. T cell (CD3^+^) activation was not observed at any time (data not shown). B cell MHC I expression also increased after RRV, I-RRV or CRW-8 stimulation for 24 h compared to unstimulated B cells (Fig. 1C, p≤0.022). MHC I levels were unaltered at 1 h and 12 h after stimulation (Figure 1C, p>0.05). Following 24 h of stimulation with RRV, I-RRV or CRW-8, B cell expression of CD86 (Figure S1A; p≤0.010) and MHC II (Figure S1B; p≤0.028) also was upregulated. On APCs, CD86 expression was increased (Figure S1A; p≤0.0017) but MHC I and MHC II levels were unaltered (data not shown). CD80 expression on APCs and B cells was unaltered by rotavirus exposure (data not shown). Therefore, exposure of NOD mouse splenocytes to rotavirus induced the activation of APCs and B cells, but not T cells. Activation of these cells was neither virus strain-specific nor replication-dependent. To determine whether RRV exposure also activated CD11c^+^ DC, the proportion of CD11c^+^ and CD11c^−^ APCs (CD3^−^CD19^−^MHCII^+^) activated after 24 h of virus exposure was assessed. Control LPS stimulation induced CD11c^+^ DC and CD11c^−^ APC activation (Figure 1D; p = 0.0001 and p = 0.0012, respectively). Stimulation with RRV, I-RRV or CRW-8 significantly increased CD69 expression on CD11c^+^ DC and CD11c^−^ APCs over unstimulated controls (Figure 1D; p≤0.0036 and p≤0.0034, respectively). Therefore, rotavirus induced the activation of DC and other APC subtypes.

### Rotavirus activation of APCs and B cells measured after 24 h of cell culture was dose-dependent and required only 1 h of rotavirus exposure

**Figure 2:**
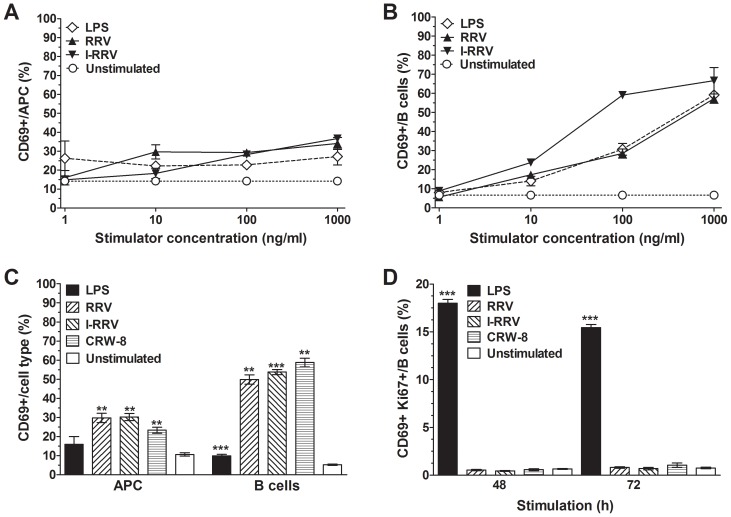
Rotavirus activation of APCs and B cells was dose-dependent and required brief virus exposure. CD69 expression on live APCs (A) and B cells (B) in NOD mouse splenocyte cultures was determined following their treatment for 24 h with the given concentrations of LPS, RRV or I-RRV. Control cultures were left unstimulated. (C) Proportions of activated live APCs and B cells after exposure of splenocytes to rotavirus, LPS or diluent (unstimulated) for 1 h, washing, replacement of medium and culture for a further 23 h. (D) Proportion of B cells expressing Ki67 and CD69 following LPS or rotavirus stimulation for 48 h and 72 h. Data are derived from one experiment and are representative of two independent experiments except for the data in D, which were derived from a single experiment. Error bars indicate the mean ± SEM of 3 replicates. ** p<0.01 and *** p<0.001 compared with the respective unstimulated control.

The concentration dependence of APC and B cell activation by rotavirus was analysed using NOD mouse splenocytes stimulated with serial dilutions of RRV or I-RRV for 24 h. Stimulation with 1 or 10 ng/ml of rotavirus did not significantly activate APCs (Figure 2A, 2B; p>0.05). APC activation was increased at 100 ng/ml of RRV or I-RRV (p = 0.0047 and p = 0.0021, respectively) and 1000 ng/ml of RRV or I-RRV (p = 0.024 and p = 0.0031, respectively). In contrast, 10 ng/ml of RRV or I-RRV was sufficient to induce B cell activation (Figure 2B, p≤0.048). Overall, APC and B cell activation was dose-dependent, with 100 ng/ml being the optimum dose (as used for the studies described above and in Figure 1).

The extent of APC and B cell activation by RRV over a large dose range of NOD mouse splenocytes was compared to RRV activation of splenocytes from C57BL/6 mice to determine if NOD mouse cells show greater sensitivity to rotavirus stimulation than those of mice not prone to autoimmunity. RRV stimulation for 24 h induced substantially more APC (Figure S2A, p<0.0001) and B cell (Figure S2B, p<0.0001) activation in NOD mouse cells over C57BL/6 mouse cells. This indicates that NOD mouse cells are more sensitive to *ex vivo* RRV stimulation than those of C57BL/6 mice.

To determine if APC and B cell activation could be induced by short-term exposure to rotavirus, splenocytes cultured with LPS, RRV, I-RRV or CRW-8 for 1 h were washed, resuspended in fresh medium and cultured for a further 23 h. Rotavirus concentrations in supernatant fluids collected at 23 h after virus removal were <0.2 ng/ml (<1×10^2^ FCFU/well). LPS stimulation for 1 h increased the proportion of activated B cells but not APCs (Figure 2C; p = 0.0071 and p>0.05, respectively). Activation by LPS was substantially less than in the earlier experiments due to the requirement for constant ligation of surface Toll-like receptor (TLR) 4 by LPS to induce optimal cellular activation. Exposure to RRV, I-RRV or CRW-8 for 1 h was sufficient to raise CD69 expression on APCs and B cells (Figure 2C; p≤0.011and p≤0.0030, respectively) after 24 h of culture. Thus, 1 h of rotavirus exposure was sufficient for APC and B cell activation. As 1 h would be sufficient for rotavirus-cell adsorption, these data suggested that rotavirus might have stimulated APCs and B cells through an intracellular mechanism.

### Rotavirus exposure did not induce B cell proliferation

Assays of intracellular Ki67 expression and ^3^H-Thymidine incorporation were employed to detect B cell proliferation in LPS- and rotavirus-stimulated splenocytes. Compared with unstimulated B cells, LPS stimulation for 48 h or 72 h increased the proportion of activated B cells undergoing proliferation (Figure 2D; p = 0.0005 and p<0.0001, respectively). In contrast, RRV, I-RRV or CRW-8 did not induce B cell proliferation (p>0.05). Similar results were observed at 44 h and 68 h when ^3^H-Thymidine incorporation was measured. LPS stimulation produced ^3^H-Thymidine uptake (mean±SEM) of 2934±445 cpm. In the absence and presence of rotavirus (RRV, I-RRV or CRW-8), 687±73 cpm and 455±40 cpm, respectively, of ^3^H-Thymidine was incorporated (p>0.05). From these findings, B cell development into antibody-secreting cells following this rotavirus stimulation *ex vivo* seemed unlikely, as typically B cell development is proliferation-dependent [25].

### Rotavirus activation of APCs and B cells was almost completely prevented by blockade of virus protein (VP) 7

**Figure 3:**
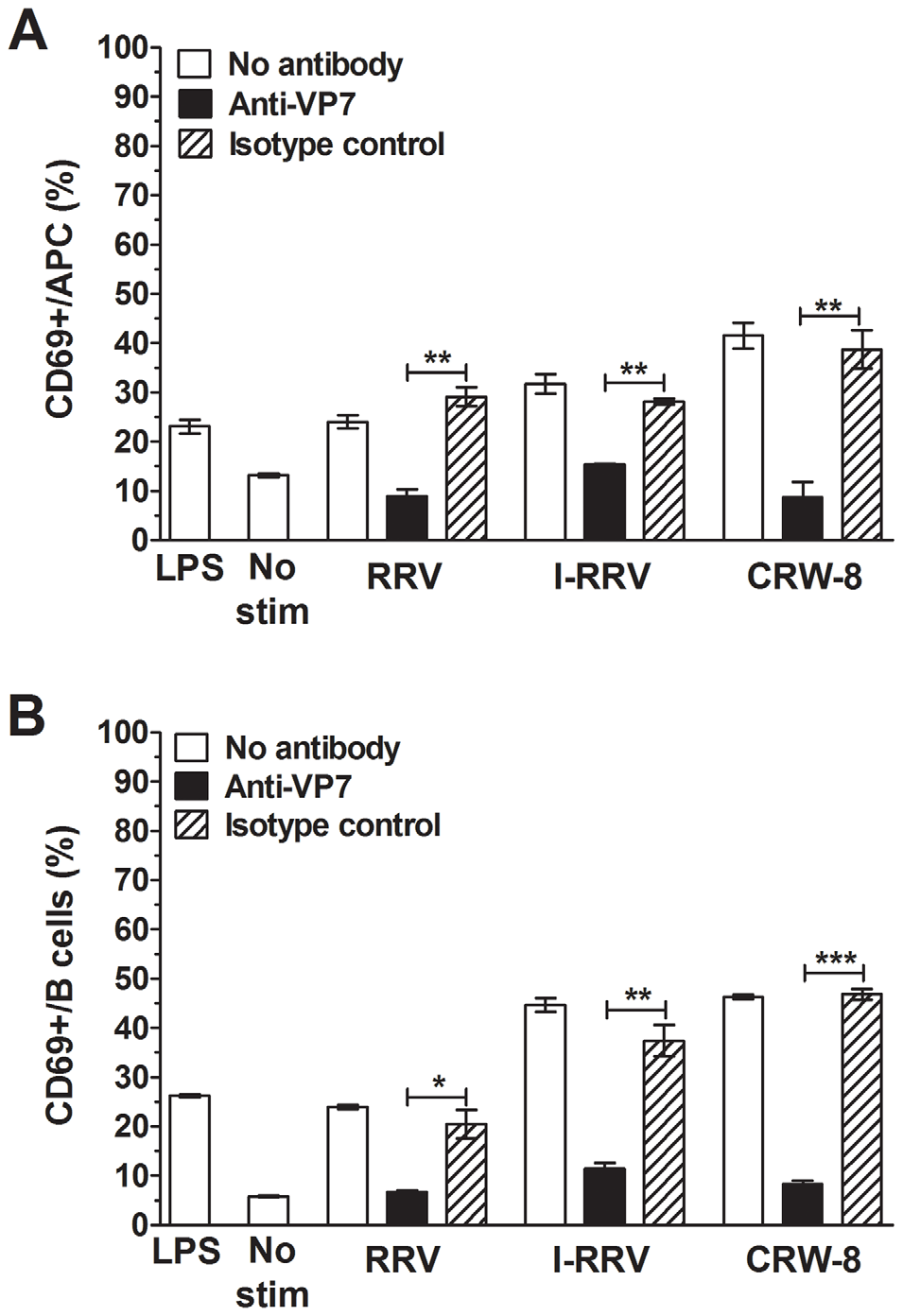
Neutralising antibody blockade of VP7 reduced APC and B cell activation. NOD mouse splenocytes were stimulated for 24/ml of rotavirus treated with anti-VP7 antibody (RV-3:1) or isotype control antibody (RV-5:2), or untreated rotavirus. CD69 expression on live APCs (A) and B cells (B) was determined by flow cytometry. Control cells were stimulated with 50 ng/ml of LPS or left unstimulated (No stim). Data are derived from one experiment and are representative of two independent experiments. Error bars indicate the mean ± SEM of 3 replicates. * p<0.05, ** p<0.01 and *** p<0.001.

Previous studies identified rotavirus outer capsid protein VP7 and rotavirus RNA as potential contributors to B cell and pDC activation [15], [18]. To investigate the importance of these rotavirus factors for NOD mouse APC and B cell activation, virions were treated an anti-VP7 antibody (RV-3:1) that neutralises rotavirus infectivity by preventing virion decapsidation and release of the dsRNA genome [26]. An isotype-matched antibody (RV-5:2) that binds human rotavirus RV-5 but not RRV or CRW-8 was reacted with virions as a control. As before, LPS activated APCs and B cells (Figure 3A, 3B; p = 0.0023 and p<0.0001, respectively), and RRV, I-RRV and CRW-8 induced CD69 expression on APCs (Fig. 3A; p = 0.0014, p = 0.0007 and p = 0.0086, respectively) and B cells (Fig. 3B; p<0.0001, p = 0.0013 and p<0.0001, respectively). Stimulation with control antibody-treated rotavirus induced similar levels of APC and B cell activation to untreated rotavirus (p>0.05). APC (Figure 3A) and B cell (Figure 3B) activation was significantly reduced following stimulation with anti-VP7-treated rotaviruses over control antibody-treated rotaviruses (p≤0.041 and p≤0.0040, respectively). The proportion of APCs and B cells expressing CD69 following stimulation with anti-VP7-treated rotavirus was similar to that in the unstimulated control (Figure 3A). Overall, antibody blockade of rotavirus VP7 prevented almost all APC and B cell activation by rotavirus. This implies that VP7 itself and/or exposure to viral RNA following virion decapsidation was required for activation of APCs and B cells.

### Activation of APCs and B cells required endosomal acidification and TLR7 signaling

**Figure 4:**
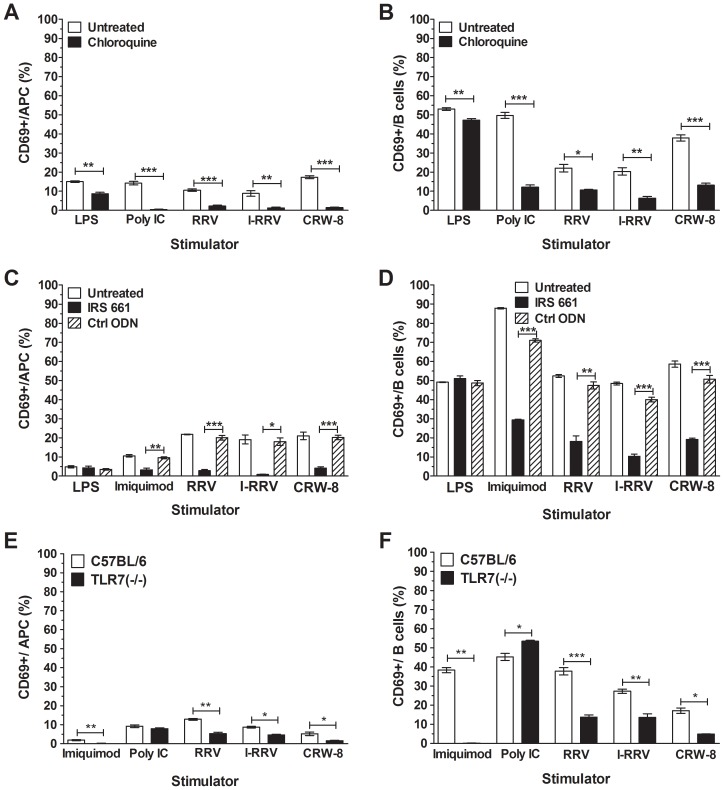
Inhibition of endosomal acidification or TLR7 signaling blocked APC and B cell activation. NOD mouse splenocytes were treated with PBS (untreated) or 10 μM chloroquine for 1 h (A, B) or with 6 μM IRS661 or Ctrl ODN for 30 min (C, D) prior to stimulation for 24 h with 100 ng/ml of rotavirus or LPS, 50 μg/ml poly IC, 1 μg/ml Imiquimod or left unstimulated. Splenocytes from C57BL/6 and TLR7 (-/-) mice were stimulated with 500 ng/ml of rotavirus, 50 μg/ml poly IC, 1 μg/ml Imiquimod or left unstimulated (E, F). CD69 expression on live APCs (A, C, E) and B cells (B, D, F) was determined by flow cytometry. The proportion of CD69-expressing cells following stimulation is shown, corrected for the proportion of background CD69 expression on unstimulated cells. Data are derived from one experiment and are representative of two independent experiments. Error bars indicate the mean ± SEM of 3 replicates. * p<0.05, ** p<0.01 and *** p<0.001 compared with the respective control.

Blockade of endosomal acidification prevents IFNα expression by pDC following RRV exposure [18]. To ascertain if endosomal acidification was necessary for NOD mouse APC and B cell activation by rotavirus, splenocytes were treated with chloroquine prior to stimulation with virus, LPS or polyinosinic:polycytidylic acid (poly IC). The proportion of activated APC and B cells in RRV-stimulated cell cultures, adjusted for the proportion activated in the absence of RRV, is shown in Figure 4. As LPS does not signal through endosomal TLRs, only a small reduction in APC and B cell activation was observed following LPS stimulation of chloroquine-treated splenocytes (Figure 4A, 4B; p = 0.0021 and p = 0.0059, respectively). In contrast, APC and B cell activation by poly IC, which signals through TLR3, was reduced when endosomal acidification was blocked (Figure 4A, 4B; p≤0.0001). Incomplete blockade of endosomal acidification or cytoplasmic receptor activation is likely to explain the residual low-level activation detected compared with unstimulated cells, which was particularly evident in B cells. Similarly, stimulation with RRV, I-RRV and CRW-8 induced CD69 expression on fewer APCs (Figure 4A; p = 0.0005, p = 0.0037 and p<0.0001, respectively) and B cells (Figure 4B; p = 0.019, p = 0.0028 and p = 0.0002, respectively) following chloroquine treatment compared to PBS-treated cells. Rotavirus and poly IC activated equivalent proportions of chloroquine-treated APCs and B cells (Figure 4A, 4B, p>0.05). Thus, blockade of endosomal acidification prevented efficient APC and B cell activation by rotavirus, indicating the likely importance of signaling through an endosomal TLR, such as TLR7, TLR9 or TLR3. As RRV-stimulated pDC contribute to B cell activation in a non-autoimmune mouse model [17] and rotavirus is a dsRNA virus, TLR7 was considered to be the most likely candidate.

To determine if signaling through TLR7 was required for APC and B cell activation by rotavirus, splenocytes were pretreated with the TLR7 antagonist, IRS661, or the control oligonucleotide, Ctrl ODN. Neither the APC nor the B cell activation following stimulation with LPS was affected by TLR7 blockade (Figure 4C, 4D, p>0.05). Conversely, treatment with IRS661 reduced APC (Figure 4C) and B cell (Figure 4D) activation following stimulation with TLR7 agonist, Imiquimod, compared to Ctrl ODN-treated cells (p = 0.0032 and p<0.0001, respectively). Similarly, fewer APC (Figure 4C) and B cells (Figure 4D) were activated by RRV (p = 0.0002 and p = 0.01, respectively), I-RRV (p = 0.016 and p<0.0001, respectively) or CRW-8 (p = 0.0003 and p = 0.0001, respectively) following IRS661 treatment compared to Ctrl-ODN treatment. The extent of rotavirus activation of IRS661-treated cells was equivalent to or less than the activation of IRS661-treated cells stimulated with Imiquimod, showing that TLR7 inhibition completely prevented APC and B cell activation by rotavirus. Overall, these data show that TLR7 recognition of rotavirus RNA is required for activation of NOD mouse APC and B cells by rotavirus.

To confirm the importance of TLR7 signalling for APC and B cell activation by rotavirus, splenocytes from TLR7 (-/-) mice (on a C57BL/6 genetic background) and C57BL/6 mice were stimulated for 24 h with Imiquimod, Poly IC, rotavirus or left unstimulated. As expected, APC and B cell activation in TLR7 (-/-) splenocytes following stimulation with Imquimod was reduced compared to C57BL/6 cells (Figure 4E, 4F; p = 0.0043 and p = 0.0013, respectively). However, no reduction in APC and B cell activation following stimulation with poly IC was observed. The proportion of APC activated by RRV, I-RRV and CRW-8 was significantly reduced in splenocytes from TLR7 (-/-) mice compared with C57BL/6 cells (Figure 4E; p = 0.0013, p = 0.010 and p = 0.019, respectively). B cell activation was similarly reduced (Figure 4F; p = 0.0004, p = 0.0068 and p = 0.0126, respectively). Interestingly, in the TLR7 (-/-) cells, the proportion of activated APC (p = 0.0036, p = 0.0009 and p = 0.01, respectively) and B cells (p = 0.0071, p>0.05 and p<0.0001, respectively) following RRV, I-RRV and CRW-8 exposure remained higher than the proportion following Imiquimod treatment. Little or no reduction in APC and B cell activation was observed in TLR3 (-/-) cells compared with C57BL/6 cells, suggesting that much of this activation does not occur through TLR3 (Figure S3). These findings indicate that TLR7 signaling is important for APC and B cell activation in non-autoimmune cells. However, in contrast to NOD mouse cells, this activation may not completely depend on TLR7 signaling.

### A soluble factor and IFNAR signaling were required for APC and B cell activation

**Figure 5:**
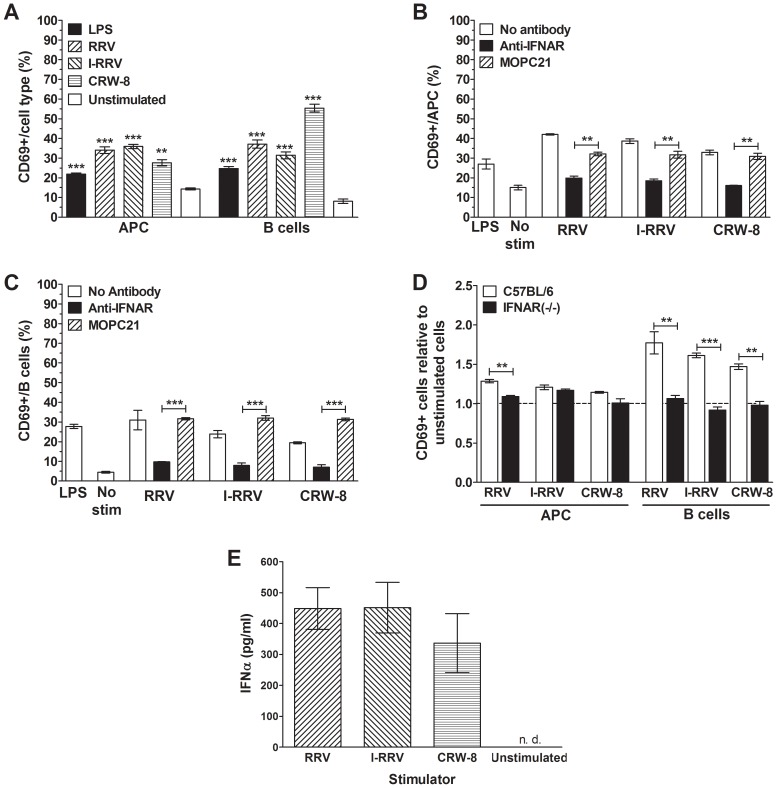
Analysis of the role of a soluble factor signaling through the IFNAR, particularly IFNα, in APC and B cell activation by rotavirus. (A) CD69 expression by APCs and B cells in NOD mouse splenocyte cultures that had been stimulated for 24 h with 150 μl of supernatant fluid collected from the experiments described in Figure 2C, together with 50 μl of RF_10_ medium. CD69 expression by APCs (B) and B cells (C) in NOD mouse splenocyte cultures treated with anti-IFNAR or isotype control antibody MOPC21 for 30 min 4°C prior to rotavirus stimulation for 24 h. Cells also were stimulated with 100 ng/ml LPS as a positive control. In (B) and (C) control cultures were unstimulated (No stim). (D) Proportions of APCs and B cells expressing CD69 in cultures of splenocytes isolated from female C57BL/6 or IFNAR (-/-) mice, after rotavirus stimulation for 24 h. Cell proportions were expressed as a ratio to unstimulated controls. (E) IFNα levels in supernatant fluids from unsorted NOD splenocytes cultured with rotavirus or left unstimulated for 24 h. Data in A to C are derived from one experiment and are representative of two independent experiments. Data in D were derived from a single experiment. Error bars in A to D indicate the mean ± SEM of 3 replicates. Data in E represent the mean ± SEM of 3 independent experiments. ** p<0.01 and *** p<0.001 compared with the respective control. n.d. =  not detected.

Exposure of human immune cells, particularly DC, to rotavirus induces the expression of multiple cytokines, including IFNα, which is capable of bystander activation [16], [17], [18], [27]. To assess the requirement for soluble factors in APC and B cell activation, supernatant fluids were collected from NOD mouse splenocyte cultures that had been treated with rotavirus for 1 h and incubated for a further 23 h. These fluids, which contained <0.2 ng/ml of rotavirus protein, were added to cultures of naive NOD mouse splenocytes for 24 h. This supernatant fluid exposure induced CD69 expression on APCs and B cells (Figure 5A; p≤0.0008 and p≤0.001, respectively). Thus, rotavirus exposure induced splenocyte production of soluble factor/s capable of activating APC and B cells.

As type I IFN has previously been shown to be important for B cell activation by rotavirus [17] the importance of IFNAR signaling for rotavirus activation of NOD-derived APCs and B cells was determined by anti-IFNAR antibody blockade. Treatment with negative control MOPC21 antibody did not significantly affect APC activation after stimulation with I-RRV or CRW-8 (Figure 5B, p>0.05). The proportion of activated APCs following MOPC21 treatment and RRV stimulation was somewhat reduced (p = 0.0091, respectively). However, the proportion of activated cells remained substantially greater after RRV stimulation than in unstimulated controls (p = 0.0072). B cell activation by rotavirus was not affected by MOPC21 treatment (Figure 5C). Blockade with IFNAR antibody prior to RRV, I-RRV or CRW-8 stimulation significantly reduced the proportion of APCs and B cells expressing CD69 compared to MOPC21-treated cells (Figure 5B, 5C; p≤0.0047and p≤0.0008, respectively). The proportions of activated APCs and B cells following stimulation of anti-IFNAR treated cells with I-RRV and CRW-8 were equivalent to unstimulated cells (Figure 5B, 5C; p>0.05). However, a small increase in B cell activation was observed following stimulation of anti-IFNAR treated cells with RRV (p = 0.0010). Therefore, signaling through the IFNAR was important for APC and B cell activation following exposure to rotavirus.

Splenocytes from IFNAR (-/-) mice (on a C57BL/6 genetic background) and C57BL/6 mice were stimulated with 100 ng/ml of RRV, I-RRV or CRW-8 for 24 h to analyse the dependence of cellular activation on signaling through IFNAR. PMA/Ionomycin C treatment as a positive control induced CD69 expression on a mean±SEM of 70±0.5% of APCs and 67±0.5% of B cells from C57BL/6 mice and 62±0.5% of APC and 41±4.7% of B cells from IFNAR (-/-) mice. Stimulation of C57BL/6 splenocytes with RRV, I-RRV or CRW-8 increased the activation of APCs and B cells relative to unstimulated cells (Fig. 5D; p≤0.0160 and p≤0.0061, respectively). As expected, the degree of activation was less than that observed in NOD cells. IFNAR (-/-) mice showed variable APC activation following stimulation with rotavirus (Figure 5D). CRW-8 stimulation did not activate APCs (p>0.05), whereas a trend for increased APC activation was seen after RRV stimulation (p = 0.0575) and I-RRV increased APC activation (p = 0.0090). The relative proportion of activated APCs following RRV stimulation was significantly reduced in IFNAR (-/-) compared to C57BL/6-derived cells (p = 0.0017). Stimulation with I-RRV or CRW-8 did not alter the activated APC proportion (p>0.05). This suggests that APC activation may not be completely dependent on IFNAR signaling. However, as the overall degree of APC activation in C57BL/6 splenocytes was not as robust as in NOD splenocytes, this interpretation is somewhat tentative. In contrast to APC, no relative increase in CD69 expression on B cells from IFNAR (-/-) mice following rotavirus exposure was observed compared to unstimulated controls (Figure 5D; p>0.05). Furthermore, the relative proportion of activated B cells following stimulation with RRV, I-RRV or CRW-8 was significantly reduced in IFNAR (-/-) compared to C57BL/6-derived splenocytes (Figure 5D; p≤0.0084). Overall, signaling through the IFNAR following rotavirus stimulation was strongly implicated in APC activation and essential for B cell activation.

To confirm that type I IFN was produced following rotavirus stimulation, IFNα levels in supernatant fluids from NOD-derived splenocytes after rotavirus exposure were determined. As expected, IFNα was not detected in unstimulated supernatant fluids (Figure 5E). In contrast, stimulation with RRV, I-RRV or CRW-8 induced IFNα secretion, at mean levels of 449 pg/ml, 451 pg/ml and 336 pg/ml, respectively. Virus strains and preparations did not differ the level of IFNα secretion induced (p>0.05). Thus, rotavirus induced type I IFN secretion in unsorted NOD splenocyte cultures.

### RRV activation of sorted immune cell populations

**Figure 6:**
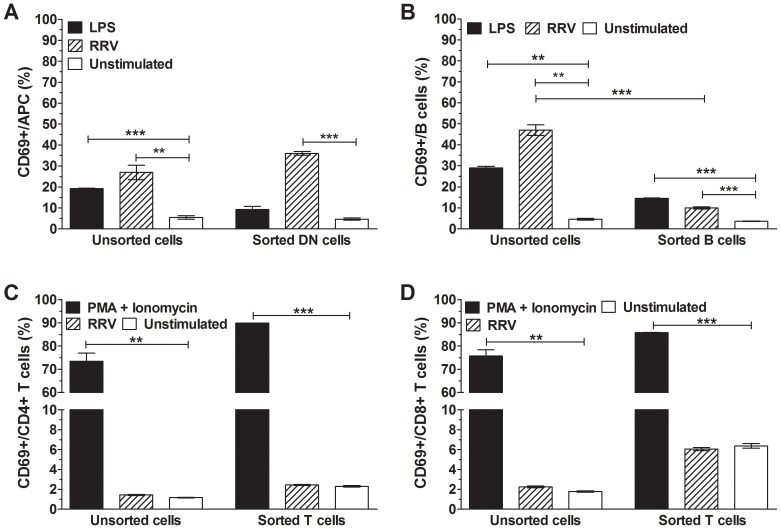
Activation of APCs, B cells, CD4^+^ T cells and CD8^+^ T cells by RRV. Sorted DN (CD3^−^CD19^−^) cells, B cells and T cells were stimulated with 100 ng/ml RRV for 24 h. The proportion of activated (CD69^+^) cells within each sorted population was determined and compared with that in unsorted cells. (A) APCs within the sorted DN population, (B) B cells within the sorted B cell population and (C) CD4^+^ and (D) CD8^+^ T cells within the sorted T cell population are shown. Control cells were stimulated with 100 ng/ml LPS (A, B) or 50 ng/ml PMA and 500 ng/ml Ionomycin C (C, D), or left unstimulated. The proportion of activated cells in unsorted splenocytes also is indicated for each cell type. Data are derived from one experiment and are representative of two independent experiments. Error bars indicate the mean ± SEM of 3 replicates. * p<0.05, ** p<0.01 and *** p<0.001 compared with the respective control.

RRV and LPS stimulation of T cells (CD3^+^CD19^−^), B cells (CD3^−^CD19^+^) and non-T and non-B cells (double negative (DN); CD3^−^CD19^−^) sorted from NOD splenocytes were assessed. The purity of these sorted DN, B cell and T cells is demonstrated in Figure S4A. LPS induced APC activation in unsorted but not DN cells (Figure 6A; p = 0.0001 and p>0.05, respectively). However, APCs in both these populations were strongly activated by RRV exposure (p = 0.0037 and p = 0.0001, respectively). Thus, RRV activated APCs in the absence of B and T cells. Additionally, IFNα was detected in supernatant fluids from DN cells stimulated with RRV, but not unstimulated DN cells (Figure S4B). LPS induced B cell activation in unsorted cells and sorted B cells (Figure 6B; p = 0.0012 and p<0.0001, respectively). Although the proportion of sorted B cells activated by RRV was increased over unstimulated controls (Figure 6B; p = 0.0002), this proportion was significantly less than that in unsorted splenocytes (p = 0.0003). This indicated that RRV was a poor activator of B cells in the absence of DN and T cells. As before, RRV stimulation of unsorted splenocytes did not induce T cell activation (Figure 6C, 6D; p>0.05). However, CD4^+^ and CD8^+^ T cells in unsorted splenocytes were activated by PMA/Ionomycin C (p = 0.0024 and p = 0.0013, respectively). Similarly, sorted T cells were activated by PMA/Ionomycin C (p<0.0001) but not RRV (p>0.05). Thus, RRV did not directly activate T cells.

### CD11c^+^ DC were necessary for lymphocyte activation by RRV

**Figure 7:**
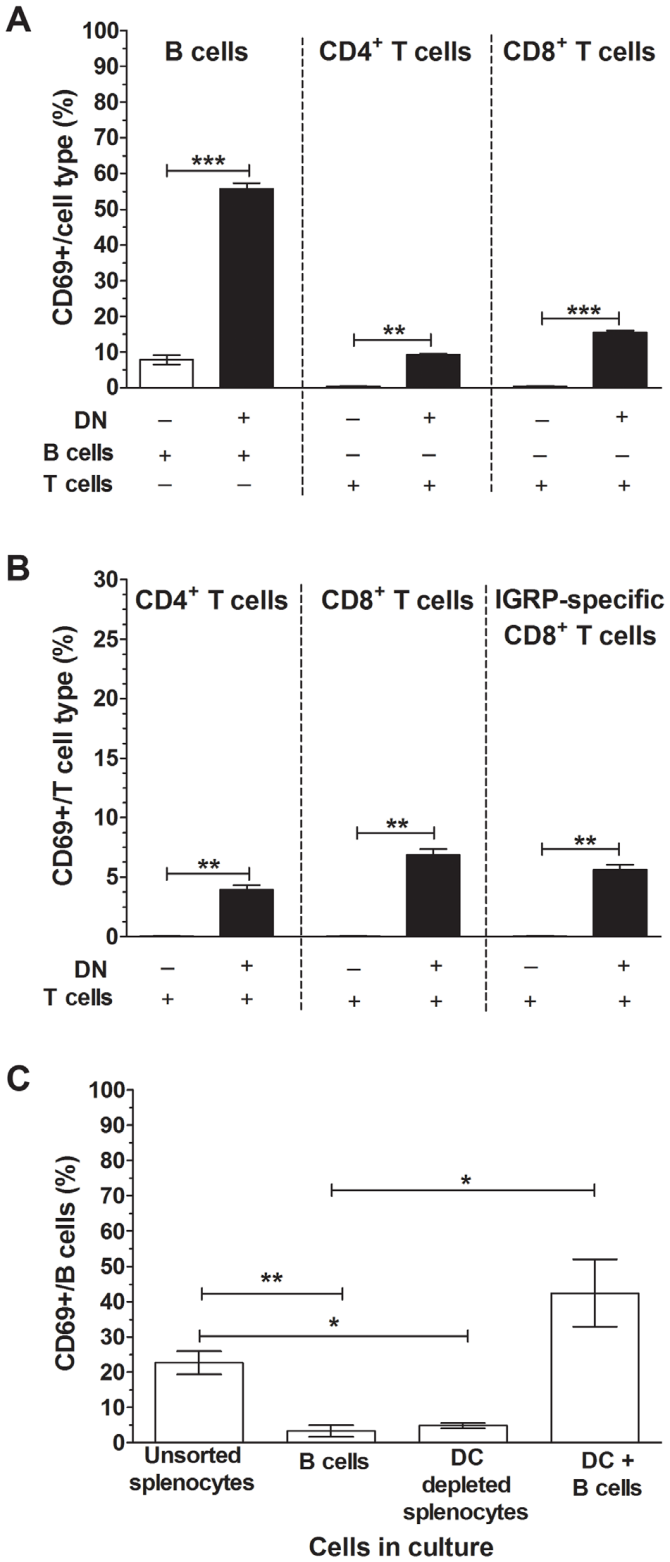
CD11c^+^ DCs were essential for lymphocyte activation by RRV. (A) Sorted DN cells (1×10^5^; CD3^−^CD19^−^) were cultured in the presence of 100 ng/ml RRV for 24 h with B or T cells (4×10^5^) sorted from NOD splenocytes (black bars). As controls, sorted B cells or T cells alone were stimulated with 100 ng/ml RRV for 24 h (white bars). The proportion of CD69-expressing B cells, CD4^+^ T cells and CD8^+^ T cells following stimulation is given, corrected for the proportion of background CD69 expression on unstimulated cells shown in Figure S5A. (B) DN cells (1×10^5^) sorted from NOD splenocytes in the presence of 100 ng/ml RRV for 24 h were cultured with T cells (4×10^5^) sorted from NOD8.3 splenocytes (black bars). As controls, T cells alone were stimulated with 100 ng/ml RRV for 24 h (white bars). The proportion of CD69-expressing CD4^+^ T cells, CD8^+^ T cells and IGRP-specific CD8^+^ T cells following stimulation is given, corrected for the proportion of background CD69 expression on unstimulated cells shown in Figure S5C. (C) Unsorted splenocytes, sorted B cells, unsorted splenocytes depleted of CD11c^+^ DC (DC depleted) and sorted CD11c^+^ DC (1×10^5^) cultured in the presence of sorted B cells (4×10^5^; DC+B cells) were stimulated with RRV as above. The proportion of CD69-expressing B cells following stimulation is given, corrected for the proportion of background CD69 expression on unstimulated cells shown in Figure S6B. Data are derived from one experiment and are representative of two independent experiments. Error bars indicate the mean ± SEM of 3 replicates. * p<0.05, ** p<0.01 and *** p<0.001.

To determine their contribution to lymphocyte activation, sorted DN cells were cultured with sorted B or T cells in the presence of RRV. The addition of DN cells in the absence of RRV induced a small increase in T cell activation, but no change in B cell activation (Figure S5A). As shown in Figure 7A, inclusion of DN cells in the presence of RRV significantly increased the proportion of activated B cells compared to sorted B cells alone (p<0.0001), and led to the activation of CD4^+^ and CD8^+^ T cells (p = 0.0011 and p<0.0001, respectively). Co-culture of sorted B and T cells activated a mean±SEM of 6.0±1.6% of B cells, equivalent to the degree of B cell activation induced by RRV stimulation of sorted B cells alone (p>0.05). Thus, rotavirus-exposed DN cells contributed to B and T cell activation. In a single experiment (data not shown), the activation of anti-IFNAR treated T cells and B cells, cultured with DN cells in the presence of RRV, was assessed. IFNAR blockade induced a similar extent of CD4^+^ T cell (0.05±0.03%), CD8^+^ T cell (0.35±0.1%) and B cell (4.6±0.7%) activation to sorted cells alone (p>0.05). Additionally, IFNα secretion was detected when T cells were cultured with DN cells and RRV, but not in cultures of T cells alone in the presence or absence of RRV (Figure S5B). This further confirms that type I IFN expression by DN cells contributes to T and B cell activation.

The ability of RRV-exposed DN cells to contribute to the activation of islet autoantigen-specific CD8^+^ T cells was assessed using T cells isolated from the splenocytes of T cell receptor-transgenic NOD8.3 mice. Most CD8^+^ T cells from these mice recognise an autoimmunity-related epitope (amino acids 206-214) from islet-specific glucose-6-phosphatase catalytic subunit related protein (IGRP). The addition of DN cells induced a small increase in T cell activation in the absence of RRV, as observed previously (Figure S5C). Culture of DN cells with T cells from these mice significantly increased the proportion of activated of CD4^+^ T cells and CD8^+^ T cells compared to sorted T cells alone in the presence of RRV (Figure 7B; p = 0.0093 and p = 0.0052, respectively). Importantly, the presence of RRV also led to the activation of IGRP-specific CD8^+^ T cells, which were detected by IGRP-specific tetramer staining (p = 0.0052). This confirms that this form of bystander activation induced by RRV results in the activation of islet-autoreactive T cells.

To determine if CD11c^+^ DC were required for B cell activation, the effect of splenocyte depletion of CD11c^+^ DC, and B cell co-culture with CD11c^+^ DC, on RRV-stimulated B cell activation was determined. The purity of the sorted CD11c^+^ DC is demonstrated in Figure S6A. As expected, the DC comprised two populations expressing variable levels of CD11c, which indicates the presence of cDC and pDC. Neither the depletion nor addition of CD11c^+^ DC increased the proportion of activated B cells in the absence of RRV (Figure S6B). However, increased background B cell activation was observed in sorted cells (Figure S6B). As expected, in the presence of RRV purified B cells alone showed less activation than B cells in unsorted splenocytes (Figure 7C; p = 0.0064). The proportion of activated B cells in splenocytes depleted of CD11c^+^ DC was similar to that in sorted B cells alone, and less than in unsorted splenocytes (p>0.05 and p = 0.035, respectively). Furthermore, CD11c^+^ DC addition to purified B cells increased B cell activation over sorted B cells alone (Figure 7C; p = 0.016). This confirmed that CD11c^+^ DC were necessary and sufficient for B cell activation following RRV exposure.

### Plasmacytoid DC, and to a lesser extent cDC, contributed to B and T cell activation by RRV

As signaling through the IFNAR and the presence of DC were shown above to be important for lymphocyte activation by rotavirus, the specific role of pDC was investigated. Sorted T and B cells were cultured alone, with cDC or with pDC, in the presence or absence of RRV for 24 h. The purity of sorted cDC and pDC, distinguished by their levels of MHC II expression, is demonstrated in Figure S7A. In all combinations, B cells were activated following stimulation with LPS and T cells were activated following stimulation with PMA/Ionomycin C, as previously shown (Figure 6). Culture of B cells with pDC but not cDC induced a small degree of activation in the absence of RRV (Figure S7B). In the absence of RRV, CD4^+^ and CD8^+^ T cells showed a small increase in activation following culture with cDC (Figure S7B).

**Figure 8:**
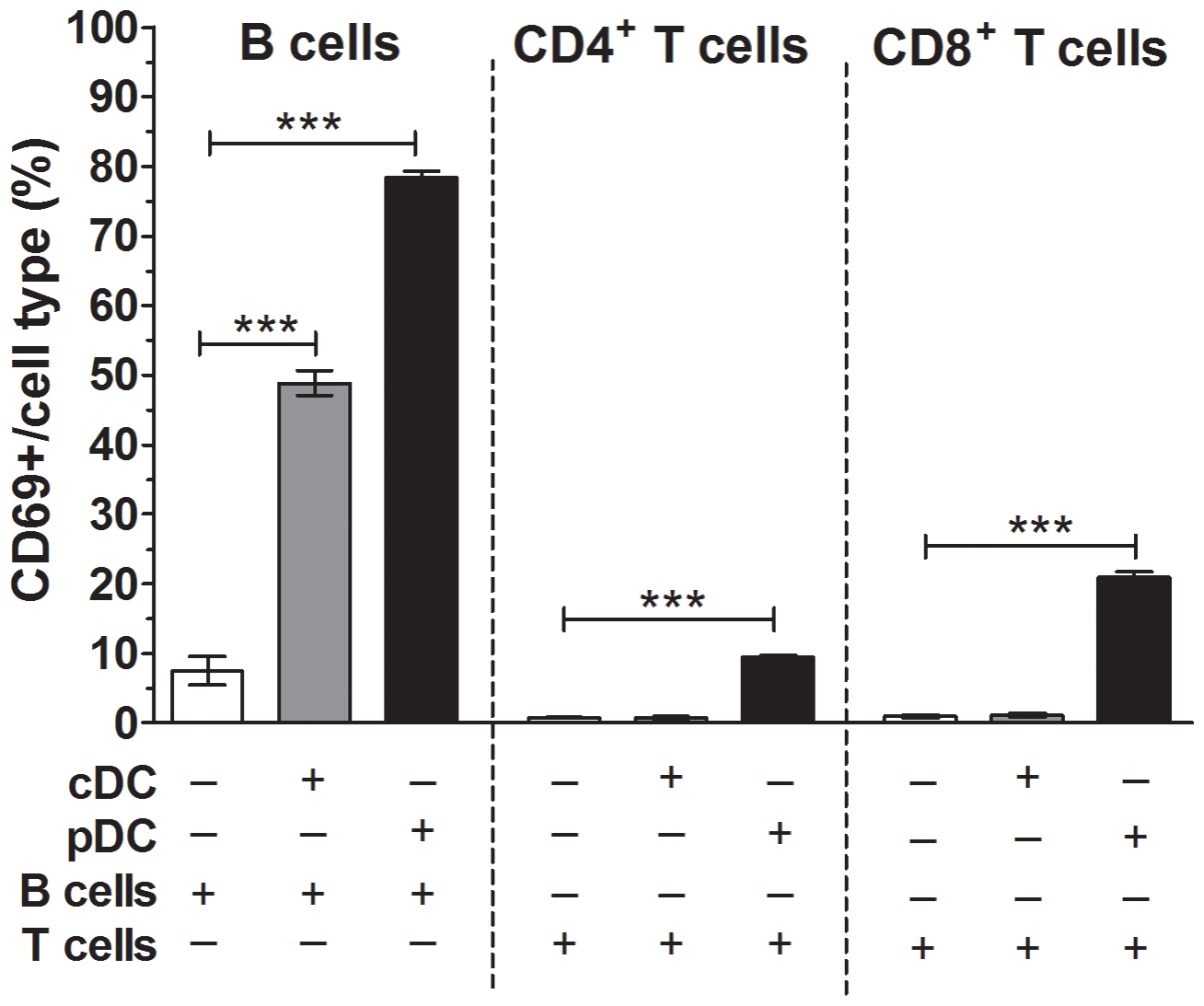
Plasmacytoid DC contributed to B cell and T cell activation by RRV. Sorted cDC (1×10^5^) (grey bars) or pDC (1×10^5^) (black bars) were cultured with sorted B or T cells (4×10^5^) in the presence of RRV for 24 h. As controls, sorted B and T cells alone were stimulated with RRV as above (white bars). The proportion of CD69-expressing B cells, CD4^+^ T cells and CD8^+^ T cells following stimulation is shown, corrected for the proportion of background CD69 expression on unstimulated cells shown in Figure S7B. Data are derived from one experiment and are representative of two independent experiments. Error bars indicate the mean ± SEM of 3 replicates. * p<0.05, ** p<0.01 and *** p<0.001.

B cells exposed to RRV were activated in the presence of both cDC and pDC (Figure 8, p = 0.0001). However, the equivalent number of pDC induced CD69 expression in a significantly larger proportion of B cells than did cDC (p = 0.0011). Thus, while both cDC and pDC contributed to B cell activation during RRV stimulation, pDC showed an enhanced ability to activate B cells. In cultures of T cells and cDC, RRV stimulation did not activate CD4^+^ or CD8^+^ T cells (Figure 8; p>0.05). In contrast, an increased proportion of CD4^+^ and CD8^+^ T cells exposed to RRV were activated in the presence of pDC (Figure 8; p<0.0001). Therefore, pDC, but not cDC, contributed to T cell activation by RRV.

Using combinations of sorted cell populations, analysis of pDC following RRV stimulation in the presence of either T or B cells showed that RRV induced activation of a mean±SEM of 15.2±2.5% pDC. In contrast, RRV stimulation of T or B cell cultures in the presence of cDC induced activation of a mean±SEM of only 2.6±1.0% of cDC. In a single experiment where cDC or pDC were cultured with T cells, RRV stimulation induced IFNα secretion in T cell cultures containing pDC but not cDC (Figure S7C). Thus, RRV activated pDC more efficiently that cDC. Overall, RRV-activated pDC contributed to the activation of B and T cells through the action of type I IFN.

## Discussion

Bystander activation is a candidate mechanism for the acceleration of type 1 diabetes development by rotavirus. Here, rotavirus stimulation of splenocytes from diabetes-prone NOD mice was shown to induce APC and B cell activation, which was prevented by VP7 blockade, inhibition of endosomal acidification and interference with TLR7 or IFNAR signaling. RRV rotavirus stimulation directly activated APCs in the absence of lymphocytes, but induced little if any activation of B and T cells in the absence of APCs. Efficient B cell activation was shown to require the presence of CD11c^+^ DC. Importantly, pDC activated both T and B cells following RRV stimulation. Exposure to RRV also induced the activation of islet-autoreactive T cells and secretion of IFNα. These findings provide strong evidence that this lymphocyte activation occurs through type I IFN expression by RRV-activated DC, mediated by recognition of rotavirus RNA. As described previously, we found that APC and B cell activation by rotavirus was independent of virus strain and replication [15], [16]. This suggests that other rotaviruses, including human and murine strains, have the potential to similarly induce activation of NOD mouse lymphocytes. Rotavirus is known to induce IFNα expression by pDC, leading to B cell activation [15], [16], [17], [18]. Similarly, depletion of pDC in human PBMC cultures reduces IFNγ expression by T cells [19]. However, to our knowledge this is the first time that type I IFN expression by pDC following rotavirus exposure has been shown to contribute to murine T cell activation, and cDC activation by rotavirus has been linked to B cell activation. In addition, TLR7 signaling is conclusively identified here for the first time as an important pathway for immune activation by rotavirus on both an autoimmune (NOD) and non-autoimmune (C57BL/6) genetic background. In C57BL/6 mice, TLR7 signaling appears to be more important for APC and B cell activation than TLR3 signaling. Our data indicate that the process of RRV-stimulated type I IFN expression by pDC leading to B cell activation is similar between NOD mice, non-diabetes prone mice and human PBMC. However, NOD mouse cells are more sensitive to activation following RRV exposure than C57BL/6 mouse cells.

Secretion of IFNα by human pDC requires rotavirus outer capsid proteins and dsRNA, which led to the proposal that virus entry (not phagocytosis) and signaling through TLR7 or TLR9 are required for this process [18]. Here we showed that VP7 decapsidation blockade and inhibition of TLR7 signaling each ablate the ability of rotavirus to induce activation of APC and B cells from NOD mice. Murine DC activation by in vitro-generated viral dsRNA fragments is TLR7-independent [28]. However, short interfering RNA has previously been shown to trigger TLR7 responses in pDC through the recognition of specific single-stranded RNA motifs [29]. By analogy with the better-studied process of rotavirus entry into epithelial cells [30], rotavirus entry into APC may involve outer capsid protein permeabilisation of the early endosomal membrane, mediated by loss of stabilising calcium ions from VP7 trimers in the low pH environment. Antibody cross-linking of VP7 inhibits this VP7 disassembly, preventing exposure of the viral dsRNA genome and genome transcription [26], [31]. Inactivated RRV retains the ability to bind VP7 antibodies and enter cells [32], [33]. As inactivated RRV also induces cellular activation, it is likely that TLR7 is triggered following dsRNA exposure in the endosome and not by replication intermediates. To our knowledge, this is the first study to demonstrate the induction of TLR7-mediated signaling by a dsRNA virus. Previously, another dsRNA virus, bluetongue, was shown to induce type I IFN expression by pDC independently of TLR7 [34]. The rotavirus activation of APC and B cells from NOD mice seems completely dependent on TLR7 signaling, whereas partial blockade of activation occurs in TLR7 (-/-) cells from C57BL/6 mice. Thus, TLR7 signaling plays a more important role in NOD than C57BL/6 cells, which may help explain the increased sensitivity of NOD cells over C57BL/6 cells to RRV stimulation. In our study, VP7 antibody blocked rotavirus infectivity through inhibition of decapsidation, so whether rotavirus binds and infects these DC or is taken up by phagocytosis cannot be determined. However, as rotavirus entry likely induces human pDC activation [18], we propose that rotavirus signaling through TLR7 in NOD mouse DC also occurs following virus entry.

**Figure 9:**
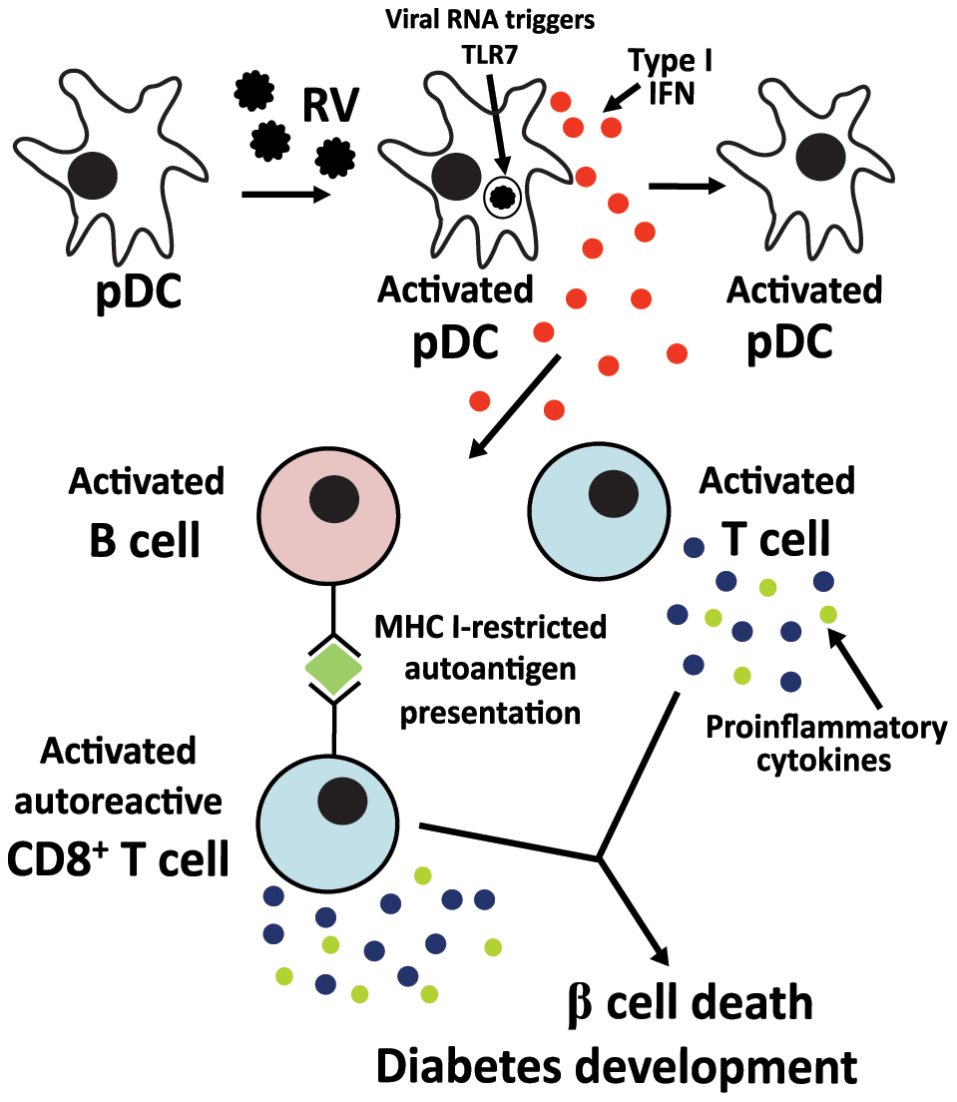
Proposed mechanism for the contribution of rotavirus-induced bystander lymphocyte activation to exacerbation of diabetes-related autoimmunity. Infectious rotavirus spreads to the PLN and MLN [14], where it infects or is phagocytosed by a minor pDC population. Within endosomes, viral dsRNA is degraded and triggers TLR7 signaling. The resulting secreted type I IFN induces the activation of bystander uninfected pDC, leading to further type I IFN release. Secreted type I IFN also induces the activation of B and T cells, including autoreactive T cells. MHC I expression on B cells is upregulated, increasing the capacity of these cells to present antigen to T cells, including autoantigen to autoreactive T cells [14]. Activated T cells increase proinflammatory cytokine expression [14]. Autoreactive T cells migrate into islets, where proinflammatory cytokine expression contributes to β cell death [60].

Studies with human pDC suggest that RRV infects a minor proportion of these cells. However, productive infection prevents IFNα production [18]. In our NOD mouse studies here, intracellular rotavirus antigen was below the limit of detection by flow cytometry (J. A. Pane, N. L. Webster and B. S. Coulson, unpublished data). This implies that productive rotavirus infection of murine splenocytes is extremely rare, and shows that very little cell-associated rotavirus is required to trigger these responses. It is likely that a small DC population is directly activated by rotavirus exposure through TLR7 signaling, while a larger population is activated by exposure to secreted type I IFN (Figure 9). This is supported by the incomplete dependence of APC activation on type I IFN signaling. Importantly, these data provide evidence that the small population of rotavirus-positive APCs detected in MLN and PLN following RRV infection of NOD mice probably would be sufficient to induce the lymphocyte activation observed in RRV-infected NOD mice [12].

RRV stimulation of unsorted NOD mouse splenocytes induced the activation of both CD11c^+^ and CD11c^−^ APCs, as expected from the RRV propensity to associate with and activate multiple small populations of NOD mouse APC subsets *in vivo* [12]. However, following *ex vivo* RRV stimulation, a trend towards increased activation of pDC over cDC activation was observed. Following RRV infection of adult NOD mice, TNF expression is increased on CD11c^+^CD8α^+^ DC, CD11c^+^ DC and CD11c^+^CD11b^+^ DC in the PLN [14]. In addition to CD11c, pDC can express CD8α and upregulate CD8α expression upon activation [35]. This suggests that the activated CD11c^+^ DC population seen *in vivo* after RRV infection of adult NOD mice may consist of pDC. As rotavirus exposure of DC subsets, including pDC, induces the expression of multiple pro-inflammatory cytokines [18], [19], [27], it is likely that the DC subsets secreting TNF in the PLN following RRV infection of NOD mice also express IFNα. While rotavirus exposure induced minimal cDC activation *ex vivo*, this was sufficient to induce B cell but not T cell activation, implying that B cells from NOD mice are highly sensitive to type I IFN-induced activation. In addition, larger proportions of B cells than T cells were activated by rotavirus, and unlike T cells, B cells were activated in unsorted splenocytes. This is further supported by previous rotavirus studies where approximately 1 pDC per 200 B cells was sufficient to induce human B cell activation [17]. Therefore, it is likely that the CD11c^+^ DC subsets other than pDC that are activated following RRV infection of NOD mice also contribute to B cell activation.

As well as inducing B and T cell activation, type I IFN also directly increases the ability of DC and B cells to present antigen to T cells [36]. Here, we showed that B cells also upregulate MHC I and CD86 expression, and APC upregulate CD86 expression, following rotavirus stimulation. This supports our findings in RRV-infected NOD mice, where PLN and MLN B cells were increased in MHC I levels and ability to induce autoreactive T cell proliferation [14]. Presentation of autoantigen by B cells on MHC I is required for diabetes development in NOD mice [37]. Additionally, B cell antigen presentation is important for T cell activation and duct injury in RRV-induced biliary atresia, which has been proposed to involve autoreactive T cell-mediated inflammation [38]. Therefore, type I IFN-mediated B cell activation may be responsible for the increased autoantigen presentation by B cells following RRV infection and contribute to diabetes acceleration. From the studies presented here, we hypothesise that upregulated type I IFN expression by DC in the lymph nodes following RRV infection of NOD mice contributes to the direct activation of autoreactive B and T cells, increased antigen presentation by B cells and induction of β cell death by cytokine-secreting autoreactive T cells (Figure 9). Therefore, type I IFN expression within the lymph nodes of RRV-infected NOD mice should be analysed in future experiments. In addition, blocking this signaling pathway *in vivo* may prevent the activation of B and T cells in NOD mice following RRV infection, as has been shown for B cell activation in IFNAR knockout mice [17].

We demonstrate a lack of B cell proliferation following rotavirus stimulation of NOD mouse splenocytes, so rotavirus exposure may not induce differentiation of these cells into antibody-secreting cells. This contrasts with human PBMC and sorted human B cells cultured with pDC, where rotavirus exposure induces B cell differentiation [16], [17]. Type I IFN-induced activation without proliferation previously has been demonstrated for murine B cells [39]. In this scenario, B cell exposure to type I IFN enhances its ability to respond to B cell receptor ligation. Human B cell proliferation following exposure to TLR7 agonists can be induced by co-culture with purified pDC, suggesting that B cell differentiation requires a greater ratio of pDC to B cells than does B cell activation [40]. Therefore, it remains possible that co-culture of purified NOD-derived pDC and B cells could lead to proliferation. However, as pDC comprise <0.5% of cells in PLN or spleen [20], it is unlikely that B cell differentiation occurs following RRV infection of NOD mice. In support of this, we have been unable to detect altered B cell proliferation in the MLN or PLN at day 7 or 14 after infection of NOD mice with RRV (J. A. Pane, N. L. Webster and B. S. Coulson, unpublished data).

Although IFNγ production is primarily associated with Th1-biased responses and contributes to IgG2a production, type I IFN also contributes to isotype switching [24]. In the absence of IFNAR signaling, influenza A virus infection induces higher local IgG1 responses, and reduced IgG2a responses [41]. Similarly, pDC depletion reduces IgA and IgG and increases IgM responses following murine rotavirus infection [17]. Therefore, the development of IgG2a-biased antibody responses in NOD mice given RRV [12] may be mediated in part by the expression of type I IFN.

In addition to increasing the ability of B cells to induce T cell activation, our data suggest that type I IFN-secreting pDC also directly induce T cell activation, including autoreactive T cells (Figure 9). Although it is likely that bystander T cell activation also occurs in non diabetes-prone mice, our data suggest that this may be at a reduced level and would be unlikely to produce autoimmune sequela. Our demonstration of autoreactive CD8^+^ T cell activation by RRV stimulation suggests that autoreactive T cells in the PLN and MLN of NOD mice would participate in this bystander activation, providing a possible explanation for the accelerated diabetes onset following RRV infection. The absence of a distinct autoreactive T cell population in RRV-infected infant NOD mice would prevent this process from occurring, which may help to clarify why they do not develop increased autoimmunity [13].

Although CRW-8 does not modulate diabetes development in NOD mice, this rotavirus induced lymphocyte activation *ex vivo*, showing that these properties are not necessarily associated. The lesser replicative ability of CRW-8, and the absence of its infectious form from the MLN and PLN, provides a possible explanation for its inability to modulate diabetes or induce B cell activation following infection of NOD mice [12], [14]. In contrast, homologous murine rotavirus is expected to replicate efficiently and spread extraintestinally in NOD mice, as occurs in other mouse strains [42]. Murine rotavirus accelerates NOD mouse diabetes development [11], and induces B cell activation *ex vivo* [15] and type I IFN-dependent B cell activation *in vivo* [17] in non-autoimmune mice. It is likely that murine rotavirus infection of NOD mice would activate lymphocytes similarly to RRV. Type I IFN signaling also contributes to B cell activation after influenza A virus infection [41]. In this case, local lung B cell responses are induced by direct signaling through the IFNAR, probably by IFNβ expression. These responses are localised to the lung and draining lymph nodes. It is conceivable that other viruses able to induce type I IFN-mediated bystander activation could augment the NOD mouse autoimmune response, providing that the bystander activation takes place in the PLN (and possibly the MLN) where autoreactive cells accumulate. In support of this hypothesis, the presentation of islet antigens in the intestine, MLN and PLN is linked [43], [44]. Gastrointestinal pathogens may have an enhanced ability to spread to the MLN and PLN from the intestine and induce type I IFN-mediated bystander activation at these sites.

Antibody blockade of the IFNAR prior to 3 weeks of age, or depletion of pDC, delays and significantly reduces the incidence of diabetes in NOD mice [20], [21]. No consistent change in pDC numbers is observed in patients with diabetes, although they show increased IFNα mRNA expression in the pancreas [45], [46], [47]. Administration of IFNα can delay diabetes development in NOD mice [48], [49]. However, IFNα expression during type 1 diabetes development is more likely to be tissue-specific and involve concomitant expression of other cytokines. Therefore, while IFNα administration may mediate diabetes protection, local type I IFN expression probably contributes to diabetes development. Our data suggest that type I IFN expression may be induced following RRV infection of NOD mice, leading to diabetes acceleration. Thus, the importance of type I IFN signaling in diabetes acceleration by RRV requires further analysis. IFNα expression following rotavirus infection of children at-risk of diabetes has not been studied. However, IFNα production appears to correlate with increased severity of initial gastrointestinal symptoms in rotavirus-infected children [50]. IFNα expression following rotavirus infection of children should be investigated in relation to their predisposition to type 1 diabetes.

Overall, these studies show that RRV induces B and T lymphocyte activation by triggering endosomal TLR7 responses in pDC and the secretion of type I IFN. Although pDC-mediated activation of bystander lymphocytes following rotavirus exposure is conserved between autoimmune and non-autoimmune mouse models, the presence of autoreactive T cells in adult NOD mice is likely to skew the outcome of this response towards increased autoimmunity. It is now important to determine if type I IFN signaling is required for diabetes acceleration by RRV. The possible use of IFNα/β levels and Th1-biased antibody responses [12] as markers of rotavirus infections likely to exacerbate islet autoimmunity in children at-risk of type 1 diabetes also should be evaluated.

## Materials and Methods

### Mice

NOD/Lt (NOD) and C57BL/6 mice were obtained from the Animal Resources Centre (Canning Vale, Western Australia). NOD8.3 TCR (NOD8.3) mice, expressing the TCRαβ rearrangements of the H-2K^d^-restricted, islet β cell-reactive CD8^+^ T cell clone NY8.3 on a NOD genetic background [51] were provided by P. Santamaria, University of Calgary, Calgary, Alberta, Canada. Mice were bred and housed in micro-isolator cages under specific pathogen-free conditions in the Biological Research Facility of the Department of Microbiology and Immunology at the University of Melbourne, as before [11], [13].

### Ethics statement

Principles of laboratory animal care' (NIH publication no. 85–23) and the ‘Australian Code of Practice for the Care and Use of Animals for Scientific Purposes (2004)’ were followed. All procedures were conducted in accordance with protocols approved by the Animal Ethics Committee of The University of Melbourne (ID 0911434).

### Viruses

Rotaviruses RRV and CRW-8 were amplified, purified by glycerol gradient ultracentrifugation and infectious titers in fluorescent cell-forming units (FCFU)/ml determined as described previously [52], [53]. Psoralen/UV inactivation of RRV was performed as before [32], [54]. The protein concentration of purified rotavirus was determined using the Bio-Rad Protein Assay.

### Murine splenocyte isolation

For analysis of unsorted cell populations, spleens were passed through 70 μm mesh, treated with red cell lysis buffer and resuspended in RPMI supplemented with 10% (vol/vol) fetal calf serum, 2 mM L-glutamine, 50 units penicillin and streptomycin (50 μg/ml) (RF_10_). For cell sorting, spleens were digested using 1 mg/ml collagenase A and 40 μg/ml DNase 1 for 30 min at room temperature prior to processing as above. Isolated cells were then reacted with 5 mM EDTA diluted in RPMI for 5 min. For sorting of cDC and pDC, splenocytes were further enriched by density-gradient centrifugation using Nycodenz (1.077 g/ml; PROGEN Biotechnik GmbH, Germany). The number of isolated cells was determined by trypan blue staining. Splenocytes isolated from IFNAR (-/-) mice on a C57BL/6 background [55] were provided by Dr Sammy Bedoui, Department of Microbiology and Immunology, The University of Melbourne. Splenocytes from TLR7 (-/-) and TLR3 (-/-) mice on a C57BL/6 background [56] were provided by Weisan Chen, Department of Biochemistry, School of Molecular Science, La Trobe University, Bundoora, Victoria, Australia.

### Sorting of leukocyte populations

For isolation of T cells (CD3^+^CD19^−^), B cells (CD3^−^CD19^+^) and DN cells (CD3^−^CD19^−^), splenocytes were stained with anti-CD3 (145-2C11)-phycoerythrin (PE) and anti-CD19 (ID3)-fluorescein isothiocyanate (FITC) diluted in RF_10_ for 30 min at 4°C and sorted using a FACS Aria (BD Biosciences). For depletion of CD3^−^CD11c^+^ DC from splenocytes and isolation of CD3^−^CD11c^+^ DC, splenocytes were stained with anti-CD3-PE and anti-CD11c (HL3)-Allophycocyanin. For isolation of pDC (CD3^−^CD19^−^MHCII^+^CD11c^+^CD45RA^+^) and cDC (CD3^−^CD19^−^MHCII^++^CD11c^++^CD45RA^−^), splenocytes were stained with antibodies to anti-CD3 (145-2C11)-PerCPCy5.5, anti-CD19 (145-2C11)-PerCPCy5.5, anti-MHCII (OX-6)-FITC, anti-CD11c-Allophycocyanin and anti-CD45RA (14.8)-PE. Population purity was >95%.

### Treatment of virus and cells

Purified anti-mouse IFNα/β Receptor 1 (BD Bioscience; IgG1, anti-IFNAR) and protein-A purified neutralising anti-VP7 monoclonal antibody RV-3:1 (IgG2b,[57]) were matched for protein concentration with purified isotype control antibodies MOPC21 (ICN Pharmaceuticals) and RV-5:2 (neutralising antibody specific for an irrelevant rotavirus strain [58]), respectively. To assess the role of VP7, rotavirus (10 ng) was incubated with 7.5 μg of RV-3:1 or RV-5:2 for 2 h at room temperature prior to culture with splenocytes. To block IFNAR signaling, splenocytes (1×10^6^) were incubated with 1 μg anti-IFNAR antibody or MOPC21 for 30 min at 4°C. Excess antibody was removed by washing prior to rotavirus exposure. For blockade of endosomal acidification, splenocytes (5×10^5^) were incubated at 37°C for 1 h prior to exposure to rotavirus with 10 μM chloroquine (Sigma, MO) diluted in PBS. For blockade of TLR7 signaling, splenocytes were treated for 30 min prior to rotavirus exposure with the synthetic oligonucleotide IRS 661 (5′-TGCTTGCAAGCTTGCAAGCA-3′) or control oligonucleotide (5′-TCCTGCAGGTTAAGT-3′) at 6 μM (Geneworks, Australia), as previously described [59].

### Stimulation of splenocytes by rotavirus

Unsorted splenocytes (5×10^5^ cells/well in U-bottomed 96-well trays) were cultured in the presence of 100 ng/ml rotavirus (unless otherwise indicated) for 1 h, 12 h or 24 h at 37°C and 5% CO_2_ (200 μl total volume). For RRV and CRW-8 rotaviruses, 100 ng/ml corresponded to 3.9 × 10^5^ FCFU/ml and 8.1 × 10^4^ FCFU/ml, respectively, equivalent to a multiplicity of infection of 0.8 and 0.2, respectively. For analysis of cellular activation following short-term rotavirus exposure, splenocytes were cultured with rotavirus for 1 h followed by washing, supernatant fluid collection and replacement with fresh RF_10_. The splenocytes were cultured for a further 23 h in the absence of rotavirus. Rotavirus infectious titres in these supernatant fluids, as determined by titration in MA104 cells, were <100 particles/well. For analysis of cell activation by secreted factors, the supernatant fluid (150 μl) of splenocyte cultures that had been stimulated with rotavirus for 1 h and cultured for a further 23 h was applied with 50 μl of fresh RF_10_ to naive, unstimulated NOD splenocytes for 24 h. Purified B, T and DN cells were stimulated with 100 ng/ml RRV alone (5×10^5^ cells/well) or in combination (at 4 B/T cells:1 DN; total 5×10^5^ cells/well) for 24 h at 37°C and 5% CO_2_. DC (pDC or cDC; 1×10^5^) cultured in the presence of B or T cells (4×10^5^) were stimulated with RRV as above. Splenocytes depleted of CD11c^+^ DC (5×10^5^ cells), and cultures of DC (2.5×10^5^ cells) with B cells (2.5×10^5^ cells), also were stimulated with RRV. As controls, cells were stimulated with *Escherichia coli* serotype 0111:B4 LPS (Sigma) at 100 ng/ml unless otherwise indicated; 50 ng/ml PMA and 500 ng/ml Ionomycin C (Sigma); 50 μg/ml poly IC (high molecular weight, InvivoGen, CA); 1 μg/ml Imiquimod (InvivoGen); or diluent. As indicated, cells or virus were pre-treated with various antibodies prior to culture, as detailed above. All cultures were performed in triplicate, unless indicated otherwise.

### Detection of cellular activation by flow cytometry

Cells were stained with the following conjugated antibodies from BD Biosciences as appropriate: anti-CD19 (ID3)-Pacific Blue, anti-CD19-FITC, anti-CD3 (500A2)-AlexaFluor700, anti-CD3-PE, anti-CD8α (53-6.7)-PerCP, anti-CD8α (53-6.7)-FITC, anti-CD4 (RM4-5)-Pacific Blue, anti-MHC II-FITC. The activation of B cells, CD8^+^ T cells (CD3^+^CD8α^+^), CD4^+^ T cells (CD3^+^CD4^+^) and APCs (CD3^−^CD19^−^MHC II^+^) was analysed with anti-CD69 (H1.2F3)-PECy7. In some experiments, APCs were further classified by CD11c expression. APC and B cells also were stained with anti-H2-K^d^ (SF1-1.1)-Biotin), anti-MHC II (OX-6)-FITC, anti-CD80 (16-10A1)-PE and anti-CD86 (GL1)-Allophycocyanin. Cells from mice with a C57BL/6 genetic background were stained with H2-D^b^ (27-14-8)-Biotin (BD) for detection of APC. Biotin was detected with Streptavadin-Allophycocyanin AlexaFluor750 (Invitrogen). For detection of IGRP-specific CD8^+^ T cells, splenocytes were stained with the IGRP_206-214_ tetramer or the negative control TUM H-2K^d^ tetramer (ImmunoID, Melbourne, Australia) as described previously [14]. Cells were stained with 7-amino-actinomycin D (Invitrogen) to exclude dead cells, as indicated. At least 100,000 cells were analysed for each sample.

### Analysis of cellular proliferation

For total proliferation measurement, unsorted splenocytes (5×10^5^ cell/well) in U-bottomed 96-well trays were stimulated with 100 ng/ml rotavirus for 44 h and 68 h at 37°C and 5% CO_2_. As controls, cells were stimulated with 100 ng/ml LPS or left unstimulated. Controls lacking cells also were included. ^3^H-Thymidine (MP Biomedicals) was added at 1 μCi/well for the final 20 h of incubation. Cells were collected onto glass fibre filters using an automated cell harvester (Skatron Instruments) and β-emission recorded by a liquid scintillation counter (Packard Bell) in counts/min. To analyse B cell proliferation, splenocytes stimulated as above for 48 h and 72 h were stained with anti-CD3, anti-CD19, anti-CD69 and anti-Ki67 (B56)-PE or mouse IgG1-PE using the eBioscience FoxP3 staining buffer kit according to the manufacturer's instructions.

### Detection of secreted IFNα

Supernatant fluids pooled from 3 replicate samples were collected from either unsorted or sorted splenocyte cultures during one or more independent experiments and stored at −80°C. The concentration of IFNα present in these pooled samples was measured with the FlowCytomix Mouse IFN-α detection kit (eBioscience).

### Statistical analysis

The Student's *t*-test, with or without Welch's correction was used. Data are derived from one experiment that was representative of the two independent experiments generally conducted. Where a single experiment only was conducted, this is indicated in the Figure legend. On graphs, error bars indicate the standard error of the mean (SEM) for replicates within a single representative experiment, unless otherwise indicated in the Figure legend. Significant differences are shown in Figures as follows: * p<0.05, ** p<0.01, *** p<0.001.

## Supporting Information

Figure S1 **Rotavirus simulation of splenocytes upregulated CD86 expression on APC and B cells and MHC II expression on B cells.** Cells (5×10^5^) isolated from 12 week-old naive female NOD mice were cultured in the presence of 100 ng/ml LPS, RRV, I-RRV, CRW-8 or left unstimulated for 24 h. (A) Surface CD86 expression (mean fluorescence intensity; MFI) on APC and B cells. (B) Surface MHC II expression (mean fluorescence intensity; MFI) on B cells. Data are derived from one experiment and are representative of two independent experiments. Error bars indicate the mean ± SEM of 3 replicates. * p<0.05, ** p<0.01 and *** p<0.001 compared with the respective unstimulated control.
(TIF)

Figure S2 **Comparison of the dose-dependence and extent of APC and B cell activation induced by RRV in splenocytes of NOD and C57BL/6 mice.** Cells (5×10^5^) isolated from naive NOD and C57BL/6 mice were cultured in the presence of given concentrations of RRV or left unstimulated for 24 h. The proportion of live APCs (A) and B cells (B) expressing CD69 was determined. Data are derived from one experiment and are representative of two independent experiments. Error bars indicate the mean ± SEM of 2 replicates. Some error bars are too small to be visible on the graph.
(TIF)

Figure S3 **Analysis of the role of TLR3 signaling in APC and B cell activation by rotavirus.** Splenocytes from C57BL/6 and TLR3 (-/-) mice were stimulated with 500 ng/ml of rotavirus, 50 μg/ml poly IC, 1 μg/ml Imiquimod or left unstimulated. CD69 expression on live APCs (A) and B cells (B) was determined by flow cytometry. The proportion of CD69-expressing cells following stimulation is shown, corrected for the proportion of background CD69 expression on unstimulated cells. Data are derived from one experiment and are representative of two independent experiments. Error bars indicate the mean ± SEM of 3 replicates. * p<0.05, ** p<0.01 and *** p<0.001.
(TIF)

Figure S4 **Stimulation of sorted DN, B and T cell populations.** (A) Splenocytes from NOD mice were sorted into populations of DN cells (CD3^−^CD19^−^), B cells (CD19^+^) and T cells (CD3^+^). A representative flow cytometry plot of each sorted population is shown. (B) Supernatant fluids pooled from 3 replicate samples of sorted DN cells cultured in the presence or absence of RRV for 24 h were assayed for IFNα using the FlowCytomix Mouse IFN-α detection kit. Error bars indicate the mean ± SEM of 3 independent experiments.
(TIF)

Figure S5 **Stimulation of B and T cells in the presence of DN cells.** (A) Sorted DN cells (1×10^5^; CD3^−^CD19^−^) were cultured with sorted B or T cells (4×10^5^) from NOD splenocytes for 24 h (black bars). Sorted B or T cells alone were cultured (white bars) as controls. (B) Supernatant fluids pooled from 3 replicate samples of sorted T cells or DN cells cultured with T cells in the presence or absence of RRV for 24 h were assayed for IFNα using the FlowCytomix Mouse IFN-α detection kit. Error bars indicate the mean ± SEM of 2 independent experiments. (C) Sorted DN cells (1×10^5^) from NOD splenocytes were cultured with sorted T cells (4×10^5^) from NOD8.3 splenocytes for 24 h (black bars). Sorted B or T cells alone were cultured (white bars) as controls. In (A) and (C), the proportion of CD69-expressing cells following 24 h of culture is shown, data are derived from one experiment (representing two independent experiments), and error bars indicate the mean ± SEM of 3 replicates. * p<0.05 and ** p<0.01.
(TIF)

Figure S6 **Analysis of B and T cell cultures lacking RRV stimulation, in the presence and absence of DC.** (A) Splenocytes from NOD mice were sorted for CD11c^+^ DC (CD3^−^CD11c^+^). A representative flow cytometry plot of this CD11c^+^ DC population is shown. (B) Unsorted splenocytes, unsorted splenocytes depleted of CD11c^+^ DC (DC depleted splenocytes) and sorted CD11c^+^ DC (1×10^5^) in the presence of sorted B cells (4×10^5^; DC+B cells) were cultured. The proportion of CD69-expressing B cells following 24 h of culture is shown. Data are derived from one experiment and are representative of two independent experiments. Error bars indicate the mean ± SEM of 3 replicates. ** p<0.01.
(TIF)

Figure S7 **Stimulation of B and T cells in the presence of cDC and pDC.** Splenocytes from NOD mice were sorted into populations of cDC (CD3^−^CD19^−^MHCII^++^CD11c^++^CD45RA^−^) and pDC (CD3^−^CD19^−^MHCII^+^CD11c^+^CD45RA^+^). A representative flow cytometry plot of each sorted population is shown. (B) Sorted cDC (1×10^5^) (grey bars) or pDC (1×10^5^) (black bars) were cultured with sorted B or T cells (4×10^5^). As controls, sorted B and T cells alone were cultured (white bars). The proportion of CD69-expressing cells following 24 h culture is shown. Data are derived from one experiment and are representative of two independent experiments. Error bars indicate the mean ± SEM of 3 replicates. * p<0.05 and ** p<0.01. (C) Supernatant fluids pooled from 3 replicate samples of sorted cDC or pDC cultured with T cells were assayed for IFNα. Data were obtained in a single experiment.
(TIF)
