# Supplementary figures and images for: Rotavirus Activates Lymphocytes from Non-Obese Diabetic Mice by Triggering Toll-Like Receptor 7 Signaling and Interferon Production in Plasmacytoid Dendritic Cells
Source: PLoS Pathog. 2014 Mar 27;10(3):e1003998. doi: 10.1371/journal.ppat.1003998 (PMC3968122; doi:10.1371/journal.ppat.1003998)

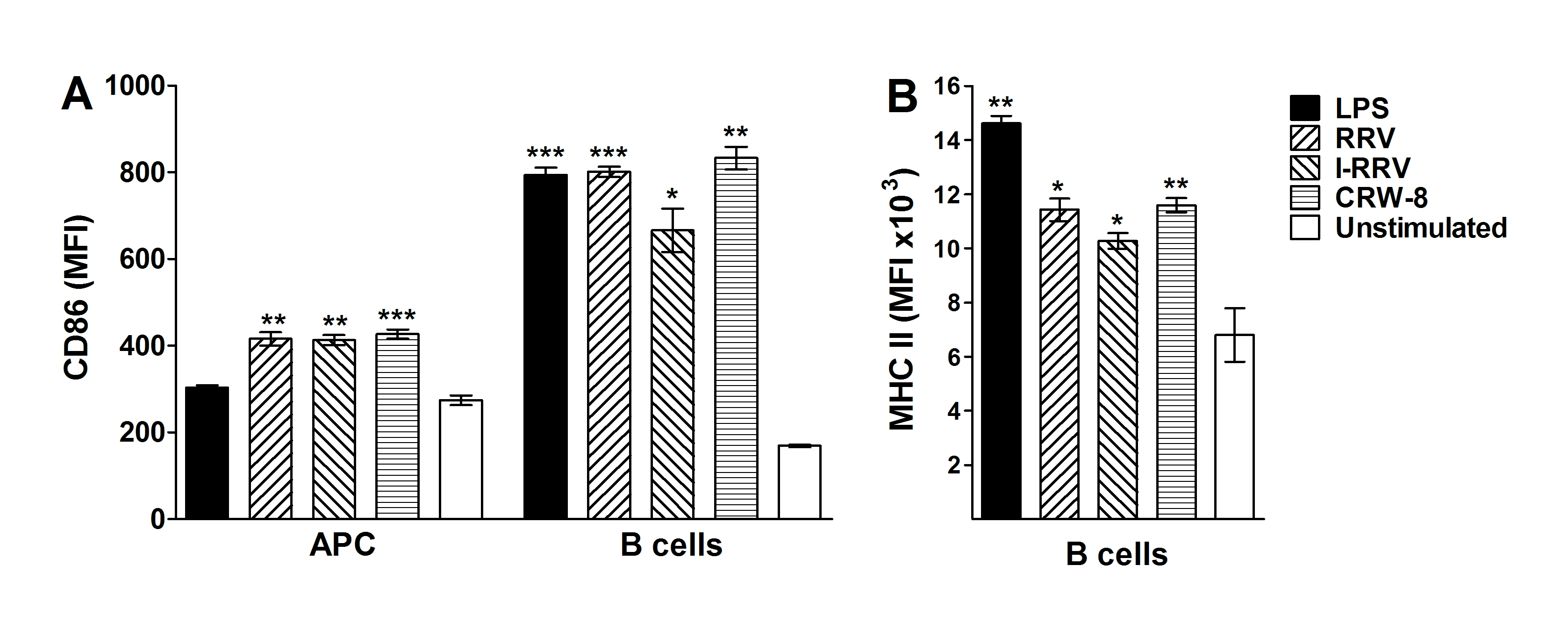

Supplement: Figure S1 — Rotavirus simulation of splenocytes upregulated CD86 expression on APC and B cells and MHC II expression on B cells. Cells (5×10^5^) isolated from 12 week-old naive female NOD mice were cultured in the presence of 100 ng/ml LPS, RRV, I-RRV, CRW-8 or left unstimulated for 24 h. (A) Surface CD86 expression (mean fluorescence intensity; MFI) on APC and B cells. (B) Surface MHC II expression (mean fluorescence intensity; MFI) on B cells. Data are derived from one experiment and are representative of two independent experiments. Error bars indicate the mean ± SEM of 3 replicates. * p<0.05, ** p<0.01 and *** p<0.001 compared with the respective unstimulated control. (TIF) [file ppat.1003998.s001.tif]

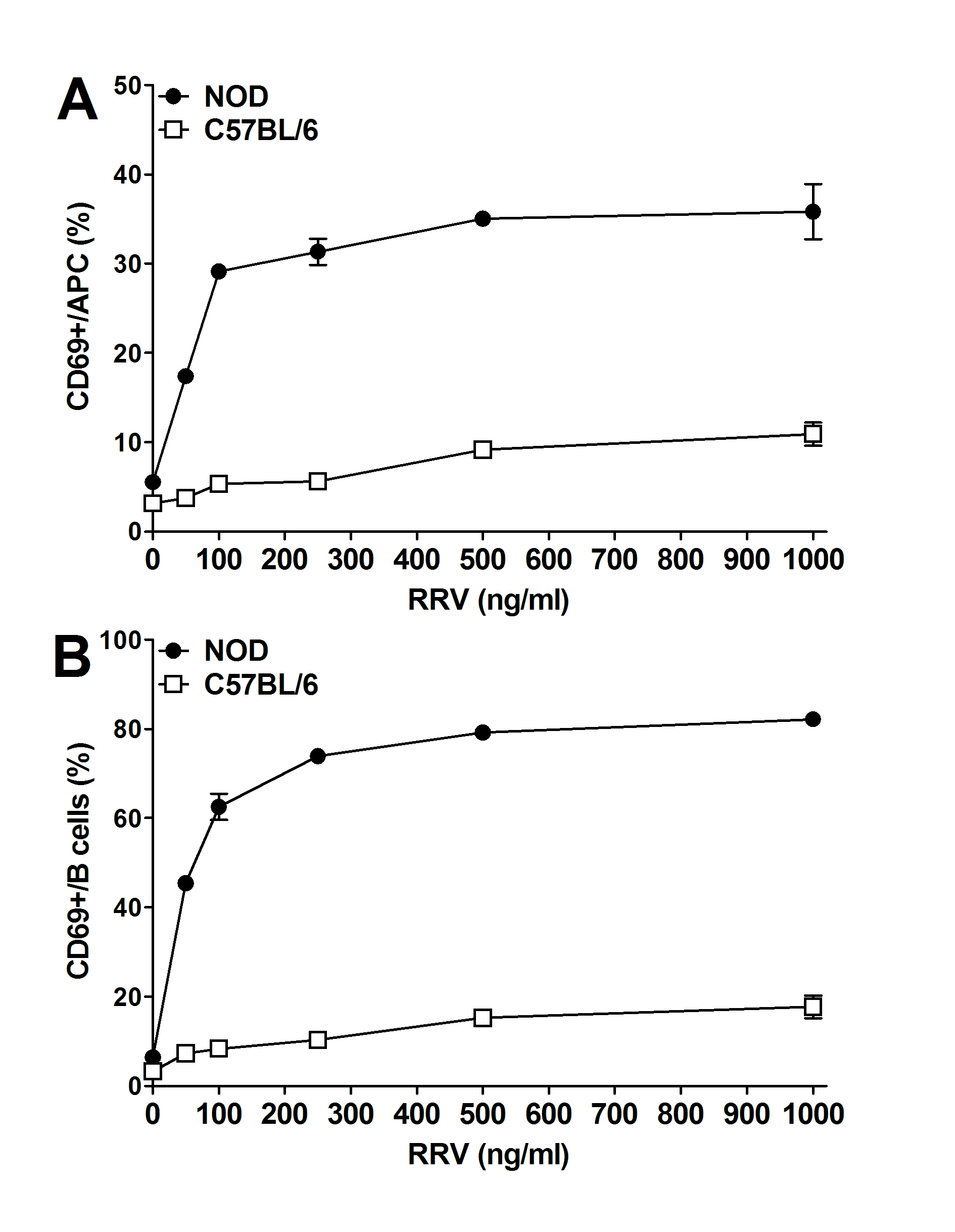

Supplement: Figure S2 — Comparison of the dose-dependence and extent of APC and B cell activation induced by RRV in splenocytes of NOD and C57BL/6 mice. Cells (5×10^5^) isolated from naive NOD and C57BL/6 mice were cultured in the presence of given concentrations of RRV or left unstimulated for 24 h. The proportion of live APCs (A) and B cells (B) expressing CD69 was determined. Data are derived from one experiment and are representative of two independent experiments. Error bars indicate the mean ± SEM of 2 replicates. Some error bars are too small to be visible on the graph. (TIF) [file ppat.1003998.s002.tif]

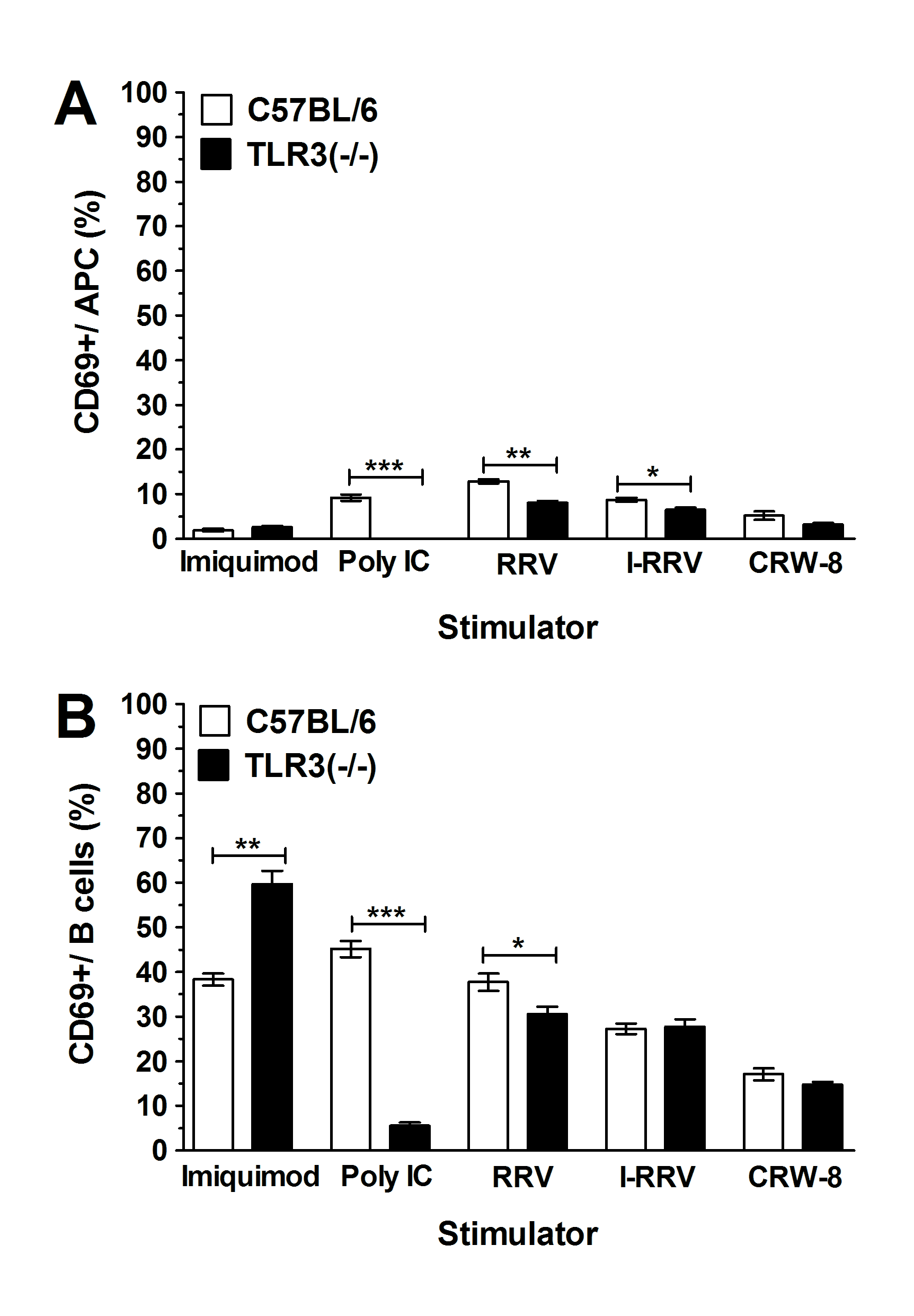

Supplement: Figure S3 — Analysis of the role of TLR3 signaling in APC and B cell activation by rotavirus. Splenocytes from C57BL/6 and TLR3 (-/-) mice were stimulated with 500 ng/ml of rotavirus, 50 μg/ml poly IC, 1 μg/ml Imiquimod or left unstimulated. CD69 expression on live APCs (A) and B cells (B) was determined by flow cytometry. The proportion of CD69-expressing cells following stimulation is shown, corrected for the proportion of background CD69 expression on unstimulated cells. Data are derived from one experiment and are representative of two independent experiments. Error bars indicate the mean ± SEM of 3 replicates. * p<0.05, ** p<0.01 and *** p<0.001. (TIF) [file ppat.1003998.s003.tif]

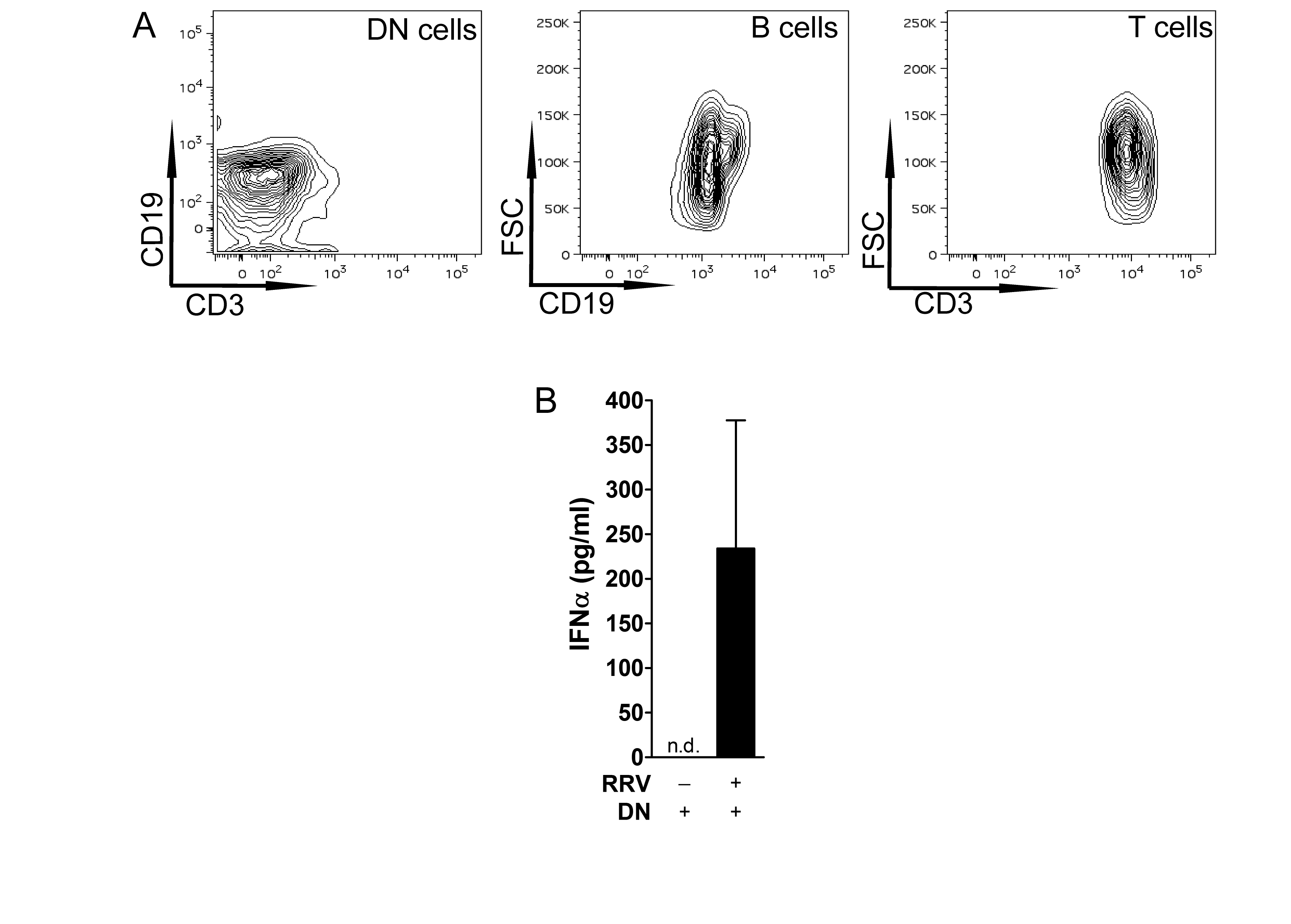

Supplement: Figure S4 — Stimulation of sorted DN, B and T cell populations. (A) Splenocytes from NOD mice were sorted into populations of DN cells (CD3−CD19−), B cells (CD19+) and T cells (CD3+). A representative flow cytometry plot of each sorted population is shown. (B) Supernatant fluids pooled from 3 replicate samples of sorted DN cells cultured in the presence or absence of RRV for 24 h were assayed for IFNα using the FlowCytomix Mouse IFN-α detection kit. Error bars indicate the mean ± SEM of 3 independent experiments. (TIF) [file ppat.1003998.s004.tif]

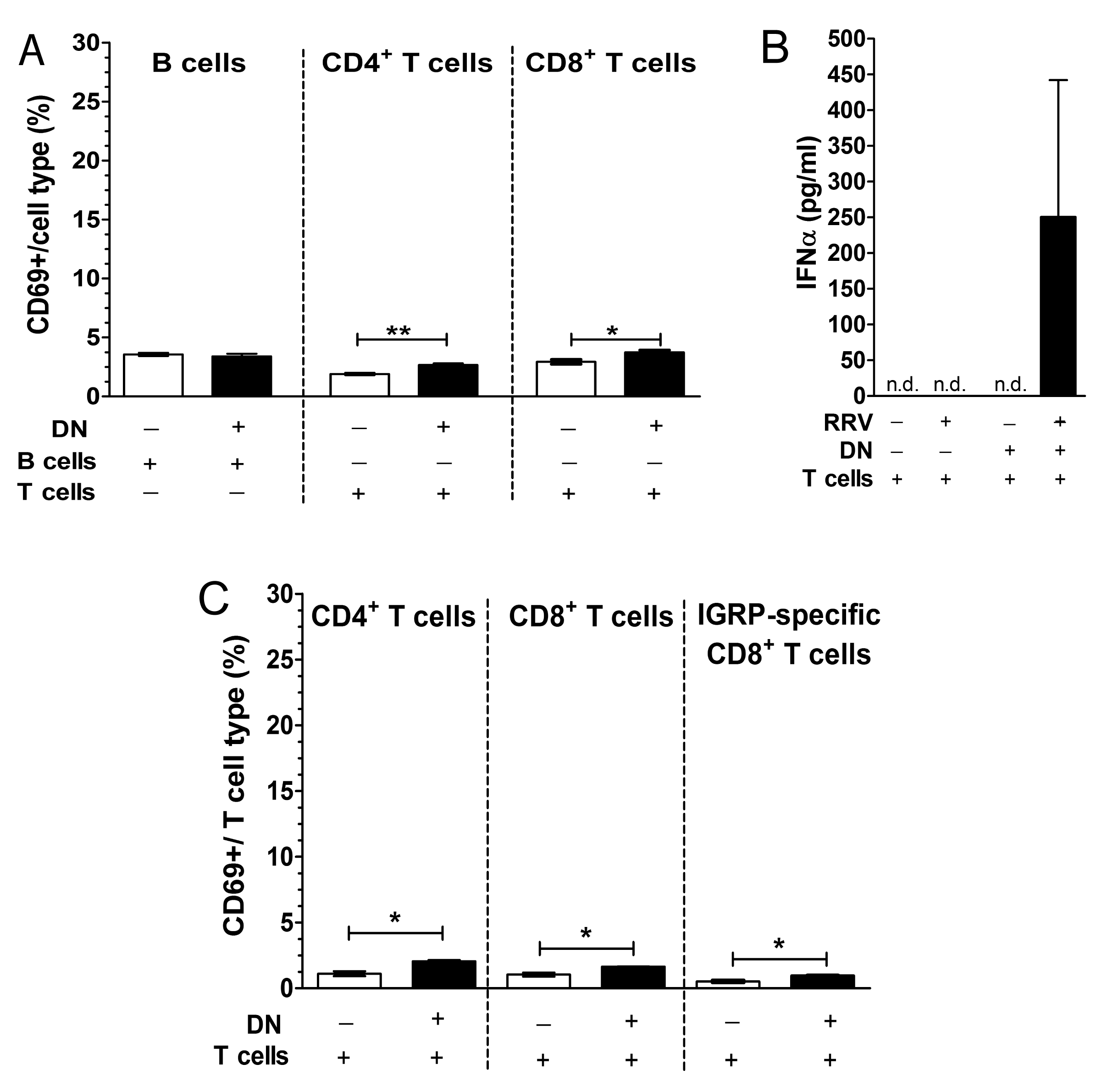

Supplement: Figure S5 — Stimulation of B and T cells in the presence of DN cells. (A) Sorted DN cells (1×10^5^; CD3−CD19−) were cultured with sorted B or T cells (4×10^5^) from NOD splenocytes for 24 h (black bars). Sorted B or T cells alone were cultured (white bars) as controls. (B) Supernatant fluids pooled from 3 replicate samples of sorted T cells or DN cells cultured with T cells in the presence or absence of RRV for 24 h were assayed for IFNα using the FlowCytomix Mouse IFN-α detection kit. Error bars indicate the mean ± SEM of 2 independent experiments. (C) Sorted DN cells (1×10^5^) from NOD splenocytes were cultured with sorted T cells (4×10^5^) from NOD8.3 splenocytes for 24 h (black bars). Sorted B or T cells alone were cultured (white bars) as controls. In (A) and (C), the proportion of CD69-expressing cells following 24 h of culture is shown, data are derived from one experiment (representing two independent experiments), and error bars indicate the mean ± SEM of 3 replicates. * p<0.05 and ** p<0.01. (TIF) [file ppat.1003998.s005.tif]

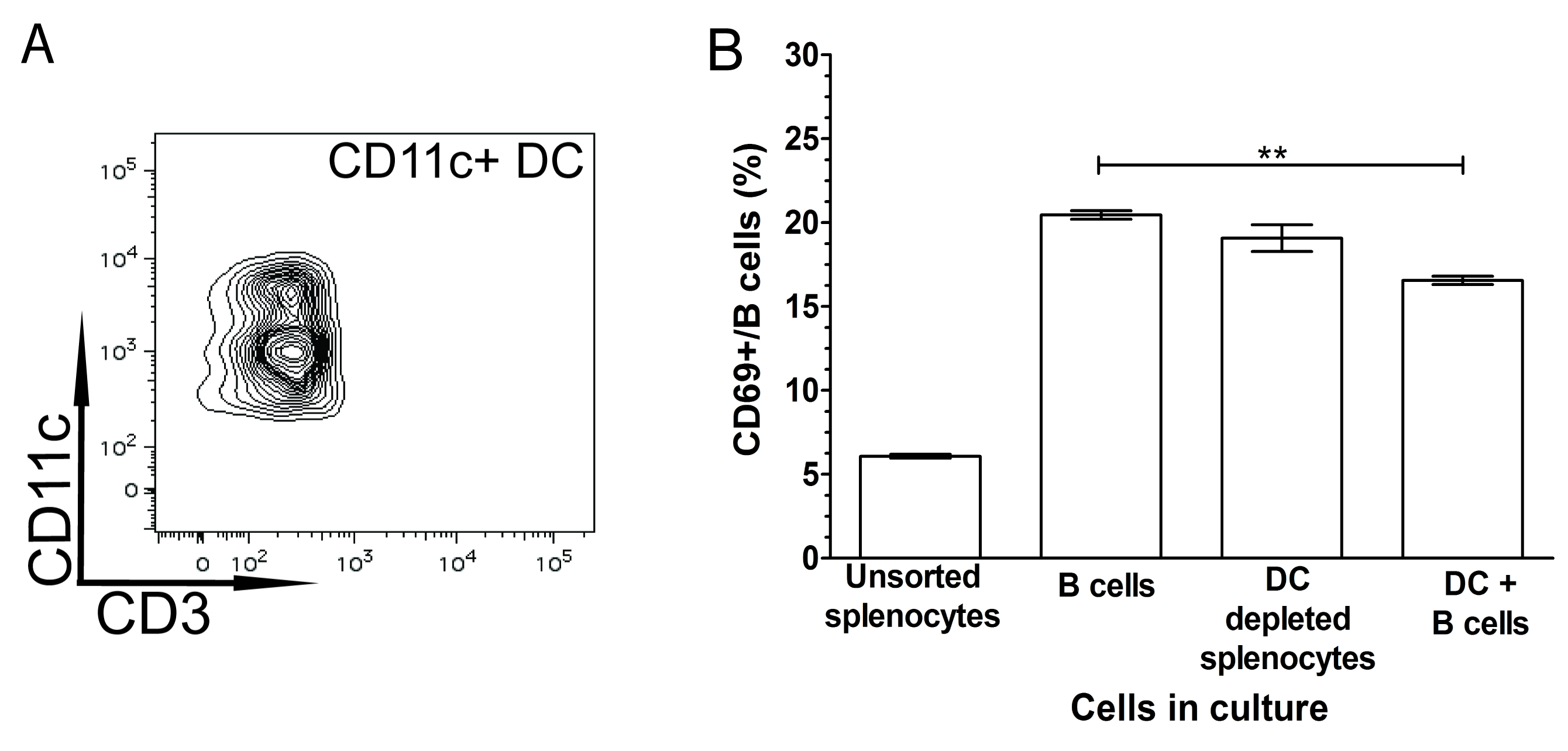

Supplement: Figure S6 — Analysis of B and T cell cultures lacking RRV stimulation, in the presence and absence of DC. (A) Splenocytes from NOD mice were sorted for CD11c+ DC (CD3−CD11c+). A representative flow cytometry plot of this CD11c+ DC population is shown. (B) Unsorted splenocytes, unsorted splenocytes depleted of CD11c+ DC (DC depleted splenocytes) and sorted CD11c+ DC (1×10^5^) in the presence of sorted B cells (4×10^5^; DC+B cells) were cultured. The proportion of CD69-expressing B cells following 24 h of culture is shown. Data are derived from one experiment and are representative of two independent experiments. Error bars indicate the mean ± SEM of 3 replicates. ** p<0.01. (TIF) [file ppat.1003998.s006.tif]

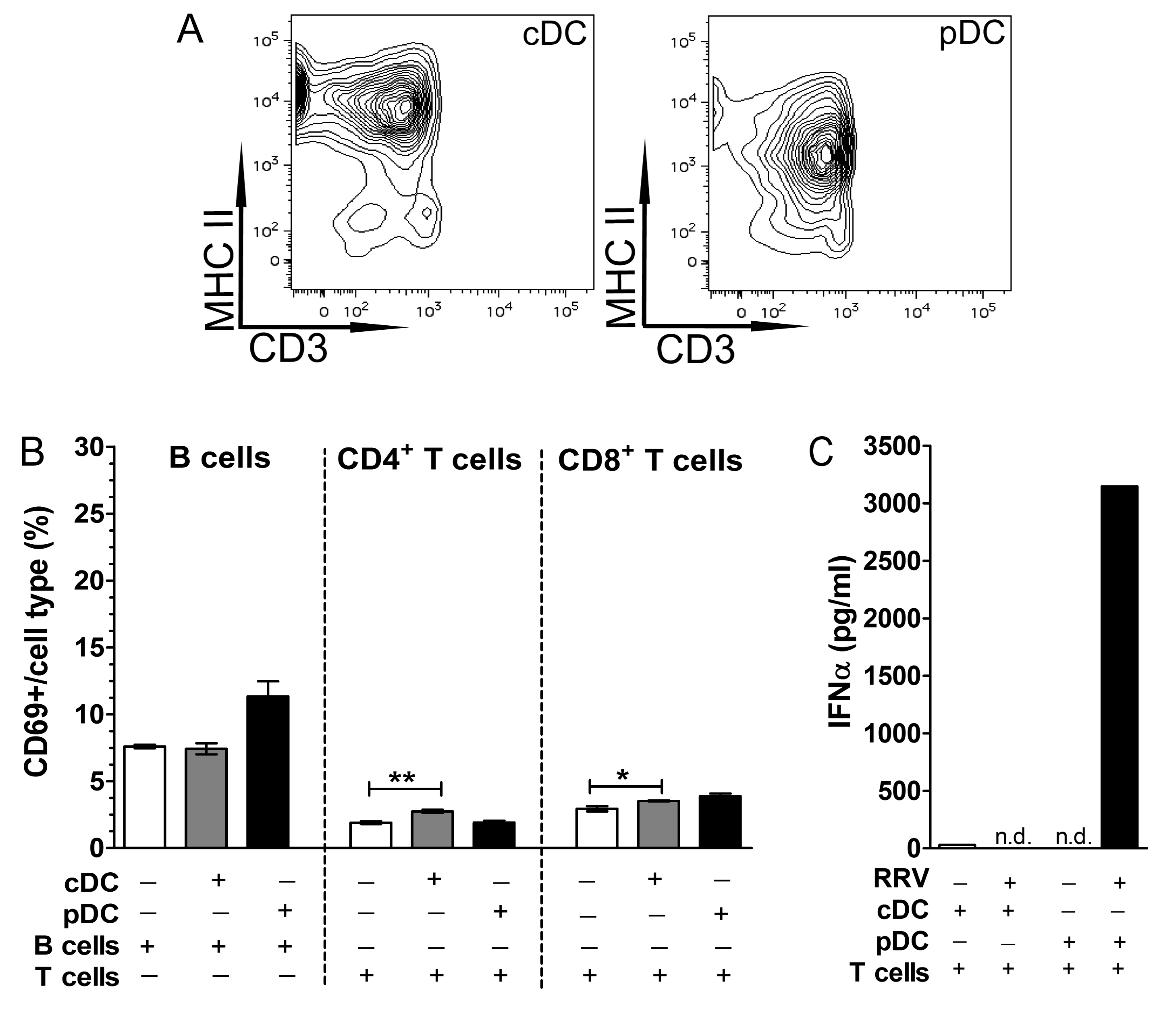

Supplement: Figure S7 — Stimulation of B and T cells in the presence of cDC and pDC. Splenocytes from NOD mice were sorted into populations of cDC (CD3−CD19−MHCII++CD11c++CD45RA−) and pDC (CD3−CD19−MHCII+CD11c+CD45RA+). A representative flow cytometry plot of each sorted population is shown. (B) Sorted cDC (1×10^5^) (grey bars) or pDC (1×10^5^) (black bars) were cultured with sorted B or T cells (4×10^5^). As controls, sorted B and T cells alone were cultured (white bars). The proportion of CD69-expressing cells following 24 h culture is shown. Data are derived from one experiment and are representative of two independent experiments. Error bars indicate the mean ± SEM of 3 replicates. * p<0.05 and ** p<0.01. (C) Supernatant fluids pooled from 3 replicate samples of sorted cDC or pDC cultured with T cells were assayed for IFNα. Data were obtained in a single experiment. (TIF) [file ppat.1003998.s007.tif]
